# Manganese salts function as potent adjuvants

**DOI:** 10.1038/s41423-021-00669-w

**Published:** 2021-03-25

**Authors:** Rui Zhang, Chenguang Wang, Yukun Guan, Xiaoming Wei, Mengyin Sha, Mengran Yi, Miao Jing, Mengze Lv, Wen Guo, Jing Xu, Yi Wan, Xin-Ming Jia, Zhengfan Jiang

**Affiliations:** 1 Key Laboratory of Cell Proliferation and Differentiation of the Ministry of Education, School of Life Sciences, Peking University, Beijing, China; 2 Peking-Tsinghua Center for Life Sciences, Peking University, Beijing, China; 3 Clinical Medicine Scientific and Technical Innovation Center, Shanghai Tenth People’s Hospital, Tongji University School of Medicine, Shanghai, China; 4 CAS Key Laboratory for Biomedical Effects of Nanomaterials and Nanosafety, National Center for Nanoscience and Technology of China, Beijing, China; 5 Laboratory for Earth Surface Processes, College of Urban and Environmental Sciences, Peking University, Beijing, China; 6 Present Address: Experimental Immunology Branch, National Cancer Institute, National Institutes of Health, Bethesda, MD USA

**Keywords:** Manganese (Mn^2+^), adjuvant, cGAS-STING, NLRP3, antigen presentation, Antigen processing and presentation, Adjuvants, Pattern recognition receptors

## Abstract

Aluminum-containing adjuvants have been used for nearly 100 years to enhance immune responses in billions of doses of vaccines. To date, only a few adjuvants have been approved for use in humans, among which aluminum-containing adjuvants are the only ones widely used. However, the medical need for potent and safe adjuvants is currently continuously increasing, especially those triggering cellular immune responses for cytotoxic T lymphocyte activation, which are urgently needed for the development of efficient virus and cancer vaccines. Manganese is an essential micronutrient required for diverse biological activities, but its functions in immunity remain undefined. We previously reported that Mn^2+^ is important in the host defense against cytosolic dsDNA by facilitating cGAS-STING activation and that Mn^2+^ alone directly activates cGAS independent of dsDNA, leading to an unconventional catalytic synthesis of 2′3′-cGAMP. Herein, we found that Mn^2+^ strongly promoted immune responses by facilitating antigen uptake, presentation, and germinal center formation via both cGAS-STING and NLRP3 activation. Accordingly, a colloidal manganese salt (Mn jelly, MnJ) was formulated to act not only as an immune potentiator but also as a delivery system to stimulate humoral and cellular immune responses, inducing antibody production and CD4^+^/CD8^+^ T-cell proliferation and activation by either intramuscular or intranasal immunization. When administered intranasally, MnJ also worked as a mucosal adjuvant, inducing high levels of secretory IgA. MnJ showed good adjuvant effects for all tested antigens, including T cell-dependent and T cell-independent antigens, such as bacterial capsular polysaccharides, thus indicating that it is a promising adjuvant candidate.

## Introduction

Vaccination is one of the most successful public health interventions. Adjuvants are used to increase the immunogenicity of antigens, with various advantages, by (1) reducing the amount of antigens; (2) reducing the number of immunizations; (3) inducing faster protection; and (4) improving the efficacy of vaccines in newborn, elderly, or immunocompromised populations.^1,2^ Over the past decades, many new adjuvants have been developed. According to their different mechanisms of action, adjuvants are divided into two categories: immune potentiators and delivery systems. Some immune potentiators composed of pathogen-associated molecular patterns (PAMPs) or synthesized activators of pattern-recognition receptors (PRRs) activate the innate immune response to induce the subsequent production of cytokines and chemokines. Other immune potentiators composed of cells or cytokines, such as dendritic cells, IL-12, or GM-CSF, directly activate immunity. Delivery systems, including liposomes, micelles, virosomes, nanoparticles, microspheres, oil/water emulsions, virus-like particles, and immune-stimulating complexes, usually carry antigens to target cells and assist antigen uptake by antigen-presenting cells (APCs).^3,4^

The immune enhancement effect of aluminum salts (Alum) was first reported by Glenny et al. in the 1920s when they accidentally found that injection of guinea pigs with diphtheria toxoid precipitated with potassium aluminum provided greater protection than with the toxoid alone.^5^ Since then, aluminum-containing adjuvants have been employed in billions of doses of vaccines and administered annually to millions of people.^6^ In fact, aluminum-containing adjuvants are the most widely used human adjuvants, partly due to their minimal reactogenicity and low cost.^7^ In addition, aluminum-containing adjuvants mainly induce T helper 2 (TH2) cell responses but not TH1 or cytotoxic T lymphocyte (CTL) responses.^1,8^ Therefore, aluminum-containing adjuvants are generally believed to be unable to elicit cellular immune responses that are essential for virus or tumor vaccines.

In recent decades, however, the medical need for new adjuvants has increased with (1) the tremendously increased use of purified antigens, such as recombinant proteins, with low immunogenicity due to the absence of immunostimulatory components recognized by PRRs and (2) the urgent need for adjuvants inducing cellular immune responses, especially CTL responses, for virus and cancer vaccines.^9–11^ To date, few adjuvants have been approved by the U.S. Food and Drug Administration for use in humans, and several formulations are in clinical trials. The oil-in-water MF59 in influenza vaccines for elderly individuals was approved in the 1990s, followed by AS03 in vaccines against avian influenza virus, AS04 in vaccines against hepatitis B virus (HBV) and human papillomavirus vaccines, and AS01 in vaccines against herpes zoster virus.^12^ Toll-like receptor (TLR) agonists, such as CpG DNA and poly (I:C), have been studied in the past two decades as new adjuvant candidates.^7^ cGAS-STING^13–16^ agonists, such as DMXAA,^17^ c-di-GMP,^18^ cGAMP,^19^ and chitosan,^20^ also showed some adjuvant effects.

Manganese (Mn) is a nutritional inorganic trace element required for a variety of physiological processes, including development, reproduction, neuronal function, and antioxidant defenses.^21,22^ Mn (Mn^2+^ in general cases) is essential for some metalloenzymes, such as Mn superoxide dismutase (SOD2, Mn^3+^ or Mn^2+^ in this case), glutamine synthetase, and arginase.^23^ However, its function in regulating immune responses is largely unknown. Previously, we found that Mn^2+^ was required for the host detection of cytosolic dsDNA by increasing the sensitivity of the DNA sensor cGAS and its downstream adapter protein STING.^24^ Importantly, Mn^2+^ was a potent innate immune stimulator by itself, inducing type I IFN and cytokine production in the absence of any infection. More recently, studies from Sohn’s lab and our lab demonstrated that Mn^2+^ directly activates cGAS independent of DNA and triggers a distinct catalytic synthesis of 2′3′-cGAMP,^25,26^ strengthening the potential applications of Mn^2+^ as a novel STING agonist to boost immune responses.

Herein, we report that Mn^2+^ promoted immune responses by facilitating antigen uptake, antigen presentation, and germinal center (GC) formation, which occurred via the activation of both the cGAS-STING and NLRP3-ASC pathways. Interestingly, induction of IL-1/18 production and release by Mn^2+^-activated inflammasomes were not observed. A colloidal manganese salt (Mn jelly, MnJ) that displayed universal adjuvant activity was produced, inducing humoral and cellular immune responses, particularly CTL activation. When administered intranasally (i.n.), MnJ also worked as a mucosal adjuvant, inducing high levels of secretory IgA. MnJ showed good adjuvant effects for all tested antigens, including T cell-independent antigens, thus indicating that it is a promising adjuvant candidate.

## Results

### Mn^2+^ promotes DC maturation via cGAS-STING activation

**Fig. 1:**
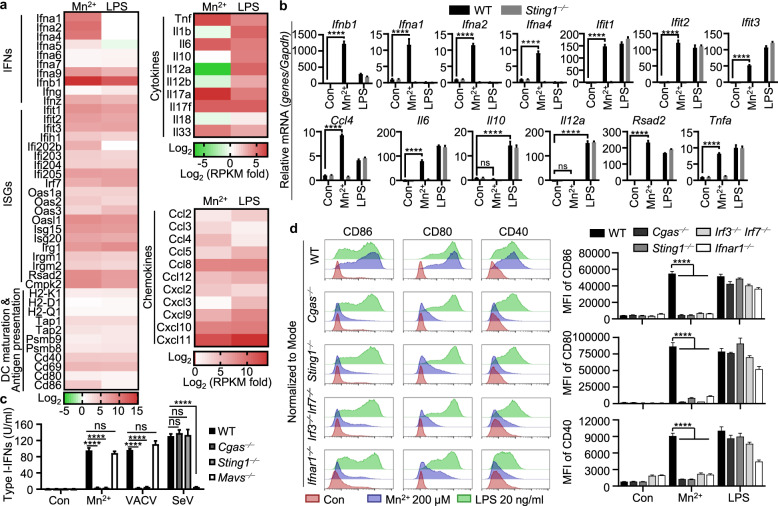
Mn^2+^ promotes DC maturation via cGAS-STING activation. **a** Heatmap of RNA-seq analysis. BMDCs were untreated or treated with MnCl_2_ (200 μM) or LPS (100 ng/ml) for 20 h. A heatmap was generated by calculating log2 ((treated RPKM)/(control RPKM)). **b** Quantitative RT-PCR analysis of the expression of the indicated gene in the WT and *Sting1*^−⁄−^ BMDCs treated with MnCl_2_ (200 μM) or LPS (100 ng/ml) for 20 h. **c** Type I IFN activity in the culture medium from WT or indicated gene-deficient BMDCs treated with MnCl_2_ (200 μM), VACV, or SeV for 24 h. **d** BMDCs from the WT or indicated gene-deficient mice were treated with the indicated concentrations of MnCl_2_ or LPS for 20 h. CD86, CD80, and CD40 expression was analyzed by FACS. One representative experiment of at least three independent experiments is shown, and each was performed in triplicate. Error bars represent the SEM; data were analyzed by an unpaired *t*-test. ns not significant; *P < 0.05; **P < 0.01; ***P < 0.001; ****P < 0.0001

Mn^2+^ is a strong type I IFN stimulator that activates the cGAS-STING pathway in the absence of infection; thus, we reasoned that Mn would help to promote the activation of adaptive immune responses. Since APCs play an important role in linking innate and adaptive immunity by processing and presenting antigens to T cells, we first determined whether Mn^2+^ promotes DC maturation. RNA-seq analysis of Mn^2+^- or LPS-treated mouse bone marrow-derived dendritic cells (BMDCs) revealed that Mn^2+^ induced robust production of both IFNβ and various IFNαs, which were not induced by LPS (Fig. 1a), together with significant upregulation of the costimulatory molecules CD80 and CD86, mouse MHC-I proteins H-2K/D/Q, immunoproteasome subunits PSMB8/9, and peptide transporters TAP1/2, and chemokines, including CCL2 and CCL3, that are able to increase the recruitment of immune cells to the injection site. Surprisingly, compared to LPS-treated BMDCs, Mn^2+^-treated BMDCs did not produce proinflammatory cytokines, including IL-1α/β and IL-18, even though the levels of TNFα and IL-6 were similar to those in LPS-treated cells, suggesting that Mn^2+^ triggered distinct signaling in BMDCs, which was confirmed by qPCR analysis (Fig. 1b). Mn^2+^ induced the secretion of type I IFNs in BMDCs in a cGAS- and STING-dependent manner but not MAVS-dependent manner (Fig. 1c). Additionally, Mn^2+^-induced expression of the costimulatory molecules CD86, CD80, and CD40 was lost in BMDCs from *Cgas*^−⁄−^, *Sting1*^−⁄−^, *Irf3*^*−⁄−*^*Irf7*^−⁄−^, and *Ifnar1*^−⁄−^ mice, showing that Mn^2+^-induced DC maturation was completely dependent on the activation of the cGAS-STING pathway (Fig. 1d).

### Mn^2+^ activates the NLRP3 inflammasome without induction of IL-1/18 production

**Fig. 2:**
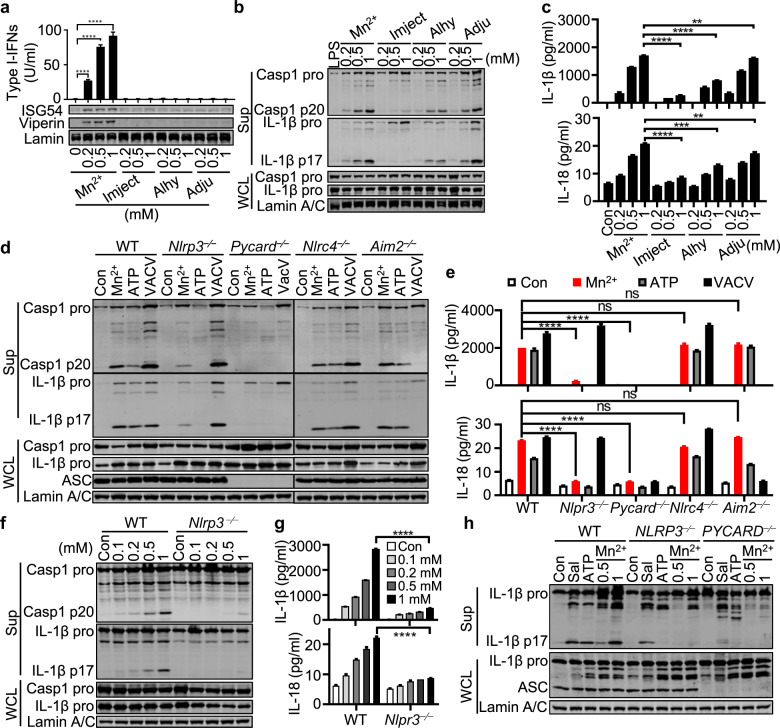
Mn^2+^ activates the NLRP3 inflammasome in murine and human macrophages. **a** Western blot (lower) and type I IFN production analyses (upper) of mouse peritoneal macrophages treated with the indicated concentrations of MnCl_2_ or aluminum salts (Imject^®^ Alum, Alhydrogel^®^ adjuvant 2%, and Adju-Phos^®^ adjuvant) for 18 h. Western blot (**b**) and ELISA analyses (**c**) of inflammasome activation in LPS-primed C57BL/6 peritoneal macrophages treated with the indicated concentrations of MnCl_2_ or aluminum salts for 5 h. Supernatants (Sup) and whole-cell lysates (WCLs) were analyzed by immunoblotting with the indicated antibodies. Western blot (**d**) and ELISA analyses (**e**) of inflammasome activation in LPS-primed WT, *Nlrp3*^−⁄−^, *Pycard*^−⁄−^, *Nlrc4*^−⁄−^, and *Aim2*^−⁄−^ peritoneal macrophages treated with MnCl_2_ (0.5 mM), ATP (5 mM), or VACV (MOI = 10). Western blot (**f**) and ELISA analyses (**g**) of inflammasome activation in LPS-primed WT and *Nlrp3*^−⁄−^ peritoneal macrophages treated with the indicated concentrations of MnCl_2_ for 5 h. **h** Western blot analysis of inflammasome activation in LPS-primed THP1 cells treated with Salmonella (Sal, MOI = 10), ATP (5 mM), or MnCl_2_ (0.5 and 1 mM). One representative experiment of at least three independent experiments is shown, and each was performed in triplicate. Error bars represent the SEM; data were analyzed by an unpaired t-test. ns not significant; *P < 0.05; **P < 0.01; ***P < 0.001; ****P < 0.0001

Many PAMPs or damage-associated molecular patterns (DAMPs) activate various inflammasomes in addition to the production of type I IFNs. Inflammasome-activated caspase-1 cleaves proinflammatory IL-1β and IL-18 into bioactive forms, which activate not only phagocytosis in neutrophils, monocytes, macrophages, and DCs but also the immune responses of Th17 and Th1 cells.^27^ Previous studies have suggested the involvement of the NLRP3 inflammasome in antibody production; however, the detailed mechanism remains controversial.^28–30^ To compare the abilities of Mn^2+^ and aluminum-containing adjuvants (Imject® Alum, Alhydrogel® adjuvant, and Adju-Phos® adjuvant) to activate innate immune responses, we tested the production of type I IFNs, IL-1β, and IL-18 in peritoneal macrophages treated with Mn^2+^ or various aluminum-containing adjuvants and found that only Mn^2+^ induced the production of type I IFNs (Fig. 2a). Mn^2+^ also more strongly activated inflammasomes than Imject® Alum and Alhydrogel® Alum (Fig. 2b, c), which was dependent entirely on ASC and largely on NLRP3 (Fig. 2d–g). Interestingly, Mn^2+^-induced inflammasome activation in THP1 cells showed complete NLRP3 dependence (Fig. 2h and Supplementary Fig. 1a). Moreover, cGAS or STING deficiency did not affect Mn^2+^-activated inflammasomes in macrophages from *Cgas*^−⁄−^, *Sting1*^−⁄−^ mice or *CGAS*^−⁄−^, *STING1*^−⁄−^ THP1 cells (Supplementary Fig. 1b–e), indicating that cGAS-STING-induced lysosomal cell death^31^ or cGAMP production^32^ was not essential for Mn^2+^ induction of inflammasome activation. Instead, we found that N-acetyl-L-cysteine (a direct scavenger of ROS), reduced L-glutathione (GSH, an intracellular thiol antioxidant), extracellular K^+^, and 2-APB (a cytosolic Ca^2+^ release inhibitor) all restrained Mn^2+^-induced inflammasome activation (Supplementary Fig. 2a–g). Using a modified culture medium, Hanks’ balanced salt solution depleted of PO_4_^3−^ and CO_3_^2−^ (herein HBSSD), in which Mn^2+^ and Ca^2+^ did not form particles, we found that Mn^2+^-activated NLRP3 and pyroptosis were essentially independent of particle formation, which is different from the case with Ca^2+^ (Supplementary Fig. 2h, i). Mitochondrial DNA depletion (Supplementary Fig. 2j) by ethidium bromide^24^ also did not affect Mn^2+^-induced inflammasome activation (Supplementary Fig. 2k, l).

Consistent with the results from RNA-seq and qPCR (Fig. 1a, b), Mn^2+^ treatment did not induce upregulation of *Il1b* and *Il18* in either murine BMDCs or human monocyte-derived dendritic cells (Mo-DCs) (Supplementary Fig. 3a). Aluminum activated the NLRP3 inflammasome in a similar way, and neither Mn^2+^ nor aluminum induced IL-1/18 production without LPS priming (Supplementary Fig. 3b, c), which was consistent with a previous report.^33,34^ The same results were obtained when human peripheral blood mononuclear cells (PBMCs) were treated with Mn^2+^ (Supplementary Fig. 3d, e). Since clinical studies on various inflammatory diseases suggested the crucial role of IL-1/18 but not of TNFα, which only amplified and perpetuated the damage,^35^ we maintained that the Mn^2+^-activated inflammasome did not cause systemic inflammation but may still be critical for its adjuvant activity (see below).

### MnJ works as a strong immune potentiator and adjuvant

Given that Mn^2+^ induced strong type I IFN production and NLRP3 inflammasome activation, both of which are implicated in promoting adjuvant activity, we reasoned that Mn^2+^ could be used as an adjuvant. To test this hypothesis, we first immunized C57BL/6 mice with ovalbumin (OVA) alone or OVA with different Mn^2+^ solutions intramuscularly (i.m.) or i.n. and measured OVA-specific antibodies. Surprisingly, we found that only Mn^2+^ in phosphate-buffered saline (PBS) but not Mn^2+^ in normal saline promoted antibody production (Supplementary Fig. 4a, b). The difference was that Mn^2+^ formed particles in PBS but not in saline, suggesting that soluble Mn^2+^ was unable to induce a local immune response, as expected. However, Mn^2+^ particles in PBS tended to aggregate and precipitate with time, thus losing adjuvant activity (Supplementary Fig. 4c, d). We generated manganese salts with different Mn^2+^/OH^−^/PO_4_^3−^ ratios and tested their immune activities. When the OH^−^/PO_4_^3−^ ratio was between 1:3 and 2:1, manganese salts were jelly like colloids (MnJ), which strongly activated both type I IFN responses and inflammasomes (Supplementary Fig. 4e and Supplementary Table 1). Manganese salts (OH^−^/PO_4_^3−^ ratio < 1:3) tended to activate inflammasomes, while manganese salts (OH^−^/PO_4_^3−^ ratio > 2:1) tended to activate only type I IFN responses (Supplementary Fig. 4e). By screening various manganese compounds, we generated MnJ (formulation #7) consisting of nanofibers with diameters < 10 nm (Supplementary Fig. 5a). MnJ was stable and without aggregation in the following experiments. Its adjuvant effect was not weakened after storage at −80 °C for 3 weeks or longer or even after freeze drying/lyophilization (Supplementary Fig. 5b). In contrast, aluminum-containing vaccines are known to be sensitive to freezing and thus hard to store or transport. Interestingly, MnJ triggered comparable inflammasome activation (Supplementary Fig. 5c) but significantly stronger type I IFN production than MnCl_2_ or Mn^2+^-PBS (Supplementary Fig. 5d), probably due to the facilitated transport into cells as nanoparticles.^36,37^ MnJ induced the secretion of type I IFNs in conventional DCs and plasmacytoid DCs, which was dependent on cGAS, STING, and IRF3/7 but not MAVS, ASC, or TLR4 (Supplementary Fig. 5e). In addition, the secretion of type I IFNs was the same in *Tlr4*^−⁄−^ DCs, indicating that there was no LPS contamination in the formulation of MnJ. Compared to MnCl_2_, which disappeared within hours, MnJ had a much longer muscle retention time of up to 8 days at the site of injection, which was similar to that of Mn^2+^-PBS particles (Supplementary Fig. 5f). Splenocytes were isolated from OVA-immunized mice and stimulated with major histocompatibility class II-binding OVA peptide I-A^b^ and MHC-I-binding peptide H-2K^b^ to compare T-cell activation. IFNγ expression induced in splenocytes from OVA-MnJ mice was higher than that induced in splenocytes from OVA-Mn^2+^ mice (Supplementary Fig. 5g). Consequently, the adjuvant effect of MnJ was significantly better than that of Mn^2+^-PBS.

**Fig. 3:**
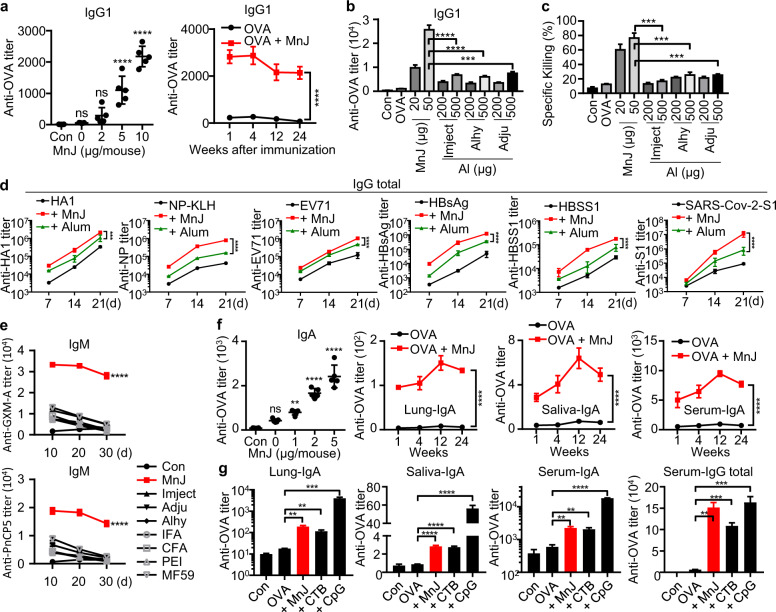
MnJ is a potent adjuvant. **a** WT mice (C57BL/6) were immunized intramuscularly with PBS or OVA (10 μg) + MnJ (0, 2, 5, or 10 μg) on days 0, 7, and 14. Sera were collected on day 21 to quantify OVA-specific IgG1 by ELISA (left, *n* = 5). Time course of OVA-specific IgG1 levels in sera from mice immunized intramuscularly three times with OVA (10 μg) + MnJ (10 μg) (right, *n* = 3). **b**, **c** WT mice (C57BL/6) were immunized intramuscularly with PBS, OVA (10 μg), OVA (10 μg) + MnJ (5 μg), or OVA (10 μg) + the indicated amounts of aluminum salts. Sera were collected on day 21 to quantify OVA-specific IgG1 by ELISA (**b**) (n = 4). OVA-specific cytotoxicity was measured on day 21 in an in vivo killing assay (**c**) (the mice were immunized on days 0, 7, and 14, *n* = 4). **d** WT mice (C57BL/6) were immunized intramuscularly with the indicated antigen (5 μg), antigen (5 μg) + MnJ (10 μg), or antigen (5 μg) + Imject Alum (1320 μg) on days 0, 7, and 14. Sera were collected on days 7, 14, and 21 to quantify HA1-, NP-, EV71-, HBsAg-, HBSS1-, or S1-specific and total IgG (*n* = 3). **e** WT mice (BALB/c) were immunized with PBS, GXM-A/PnCP5 (40 μg) alone or with MnJ (200 μg)/Imject Alum (1000 μg)/Alhydrogel (1000 μg)/Adju-Phos/IFA (50 μl)/CFA (50 μl)/PEI (100 μg)/MF59 (50 μl) on days 0, 10, and 20 through multipoint injection (intramuscularly, subcutaneously, and intraperitoneally). Sera were collected on days 10, 20, and 30 to quantify GXM-A/PnCP5-specific IgM by ELISA (*n* = 4). **f** WT mice (C57BL/6) were immunized intranasally with PBS or OVA (10 μg) + MnJ (0, 1, 2, or 5 μg) on days 0, 7, and 14. Sera were collected on day 21 to quantify OVA-specific IgA by ELISA (left, *n* = 5). Time course of OVA-specific IgA levels in BALF, saliva, and serum, as indicated, from mice immunized intranasally three times with OVA (10 μg) + MnJ (5 μg) (right, *n* = 3). **g** OVA-specific and total IgA and IgG levels were measured by ELISA on day 21 after intranasal immunization with OVA (10 μg), OVA (10 μg) + MnJ (5 μg), OVA (10 μg) + CTB (10 μg), or OVA (10 μg) + CpG (1 μg) on days 0, 7, and 14 (*n* = 3). One representative experiment of at least three independent experiments is shown, and each was performed in triplicate. Error bars represent the SEM; **a**, **d**, **e**, **f** data were analyzed by two-way ANOVA; **a**, **b**, **c**, **f**, **g** data were analyzed by an unpaired *t*-test. ns not significant; *P < 0.05; **P < 0.01; ***P < 0.001; ****P < 0.0001

We next evaluated the adjuvant effect of MnJ in detail. MnJ-adjuvanted induction of antibody production by intramuscular immunization exhibited dose dependence and lasted for at least 6 months (Fig. 3a). Compared to three different aluminum-containing adjuvants, MnJ induced much stronger OVA-specific IgG1 production and CTL responses (Fig. 3b, c). We also compared MnJ with other adjuvants, including complete Freund’s adjuvant (CFA), incomplete Freund’s adjuvant (IFA), MF59, and polyetherimide (PEI), and found that the MnJ adjuvant effect was even better than that of CFA (20 μg of MnJ vs 50 μl of CFA, i.m.) and MF59 (20 μg of MnJ vs 50 μl of MF59, i.m.) (Supplementary Fig. 5h, i). Additionally, MnJ boosted specific antibody production against different recombinant protein/peptide antigens, including influenza A/PR8 hemagglutinin A1 peptide, haptenized experimental antigen nitrophenol-conjugating keyhole limpet hemocyanin, HBV surface antigen (HBsAg) and HBSS1 fusion protein (containing S (1-223 aa) and PreS1 (21-47 aa)),^38^ the S1 subunit protein (14-685 aa) of SARS-CoV-2, and inactivated enterovirus type 71 (EV71) (Fig. 3d). Bacterial capsular polysaccharides (CPs) are generally believed to be T cell-independent type 2 (TI-2) antigens that mainly activate B cells, resulting in the production of IgM due to the absence of isotype switching.^39,40^ The adjuvant effect of MnJ on the induction the production of IgM against two bacterial CPs, glucuronoxylomannan-A (GXM-A) from *Cryptococcus neoformans*^41^ and pneumococcal capsular polysaccharide serotype 5 (PnCP5), was also tested. We found that MnJ strongly promoted the production of CP-specific IgM, while other tested adjuvants showed weak or no adjuvanticity to CP (Fig. 3e). In addition, intranasal immunization revealed that MnJ was also a potent mucosal adjuvant, inducing IgA antibody levels in the lung, saliva, and serum for a long time that were as good as those induced by cholera toxin B (Fig. 3f, g).

Importantly, consistent with no induction of proinflammatory cytokine IL-1/18 production in Mn^2+^-treated cells, compared to CFA-injected mice showing prominent swellings and granulomas with one injection (Supplementary Fig. 5j), MnJ-injected mice displayed no visible side effects on the injection site, body weight, survival, or different organs even after repeated administrations (three injections in 3 consecutive weeks) (Supplementary Fig. 5k, l), suggesting that MnJ is a safe adjuvant with good biocompatibility. Importantly, draining lymph nodes (dLNs) in MnJ-injected mice showed significantly increased volumes and weights, indicating that MnJ promoted the migration and/or proliferation of immune cells (Supplementary Fig. 5l, far right, enlarged).

### MnJ promotes antigen presentation and T-cell responses

**Fig. 4:**
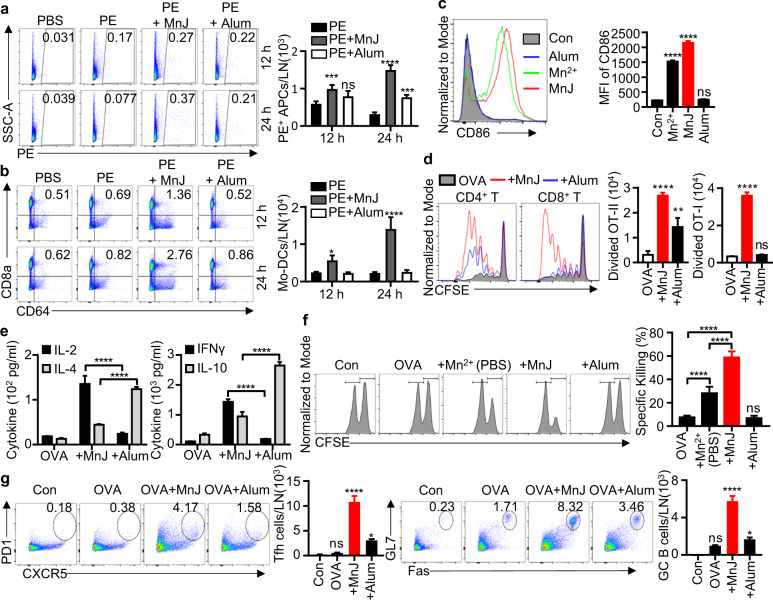
MnJ promotes antigen presentation and specific T-cell responses. **a**, **b** WT mice were immunized subcutaneously with PBS, phycoerythrin (PE, 10 μg), PE (10 μg) + MnJ (20 μg), or PE (10 μg) + Imject Alum (200 μg). Inguinal lymph nodes were collected 12 or 24 h later (*n* = 3). The ratio and number of PE^+^ APCs (**a**) and Mo-DCs (**b**) among dLN cells were analyzed by FACS. Live cells were identified by DAPI staining. Among live singlet cells, APCs were identified as the cell subset single or double positive for CD11c and F4/80. Among APCs, Mo-DCs were identified as CD8a^+^ CD64^+^ cells. **c** BMDCs were treated with MnCl_2_ (20 μg/ml), MnJ (20 μg/ml), or Imject Alum (20 μg/ml) for 20 h. CD86 expression was analyzed by FACS. **d** CD45.1^+^ OT-I CD8^+^ or OT-II CD4^+^ T cells were labeled with CFSE and transferred to CD45.2^+^ WT mice. These mice were then immunized with OVA (1 μg), OVA (1 μg) + MnJ (10 μg), or OVA (1 μg) + Imject Alum (1320 μg). After 3 days, T-cell proliferation was analyzed by FACS (*n* = 3). **e** WT mice were immunized intramuscularly with OVA (10 μg), OVA (10 μg) + MnJ (10 μg), or OVA (10 μg) + Imject Alum (1320 μg) on days 0, 7, and 14. Splenocytes were collected on day 21 and stimulated with OVA (100 μg/ml). IL-2, IL-4, IFNγ, and IL-10 secretion by T cells was measured by ELISA (*n* = 3). **f** OVA-specific cytotoxicity was measured on day 21 in an in vivo killing assay (mice were immunized on days 0, 7, and 14, *n* = 4). **g** Numbers of Tfh or GC B cells in dLNs from WT mice were analyzed by FACS. Live cells were identified by DAPI staining. Among live singlet cells, CD4^+^ T cells were identified as the cell subset double positive for CD3 and CD4. Among CD4^+^ T cells, Tfh cells were identified as PD1^+^ CXCR5^+^ cells. B cells were identified as the cell subset double positive for CD45 and B220. Among B cells, GC B cells were identified as Fas^+^ GL7^+^ cells (*n* = 3). One representative experiment of at least three independent experiments is shown, and each was performed in triplicate. Error bars represent the SEM; data were analyzed by an unpaired *t*-test. ns not significant; *P < 0.05; **P < 0.01; ***P < 0.001; ****P < 0.0001

MF59 and aluminum-containing adjuvants facilitate APC engulfment of antigens and transport to dLNs, where they induce the differentiation of monocytes to dendritic cells.^42,43^ We next evaluated antigen uptake by APCs and Mo-DC differentiation in dLNs after MnJ administration. We immunized mice in inguinal regions subcutaneously with the fluorescent protein phycoerythrin (PE) containing MnJ or an aluminum-containing adjuvant. APCs in inguinal lymph nodes were analyzed by flow cytometry afterward. The percentage and number of PE-loaded APCs were significantly enhanced 12 and 24 h after MnJ immunization (Fig. 4a and Supplementary Fig. 6a). Additionally, there was a significant increase in the accumulation of Mo-DCs in mice immunized with antigen plus MnJ compared to that in mice immunized with antigen plus aluminum-containing adjuvant or alone (Fig. 4b and Supplementary Fig. 6a).

The capacity of MnJ to promote BMDC maturation was stronger than that of Mn^2+^, while aluminum-containing adjuvants did not have any effect (Fig. 4c). In vivo, MnJ enhanced both CD4^+^ and CD8^+^ T-cell proliferation, whereas aluminum induced only weak CD4^+^ T-cell proliferation (Fig. 4d). Splenocytes were next isolated from OVA-immunized mice and stimulated with OVA to compare T-cell activation. IL-2 and IFNγ production were highly induced in splenocytes from OVA-MnJ-immunized mice but not in those from OVA-aluminum-immunized mice, whereas IL-4 and IL-10 were preferentially produced via OVA-aluminum immunization (Fig. 4e), indicating that MnJ potently stimulated the TH1 response in addition to the TH2 response. Accordingly, an in vivo cytotoxic assay showed that MnJ-immunized mice generated very strong CTL activities in killing OVA-bearing cells, which was absent in aluminum-immunized mice (Fig. 4f). MnJ also promoted the formation of GCs with significantly increased amounts of Tfh and GC B cells (Fig. 4g and Supplementary Fig. 6b). Therefore, MnJ promoted immune responses by facilitating antigen uptake, antigen presentation, and GC formation. Collectively, these results indicated that MnJ functions as a good adjuvant by acting as both an immune activator and a delivery system.

### Both cGAS-STING and the NLRP3 inflammasome contribute to the adjuvant activity of MnJ

**Fig. 5:**
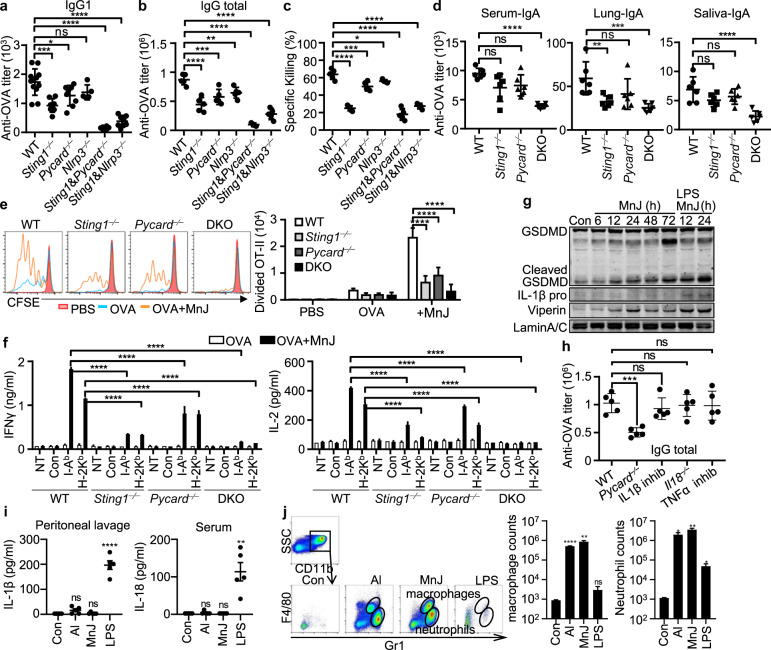
Both cGAS-STING and NLRP3 contribute to the adjuvant activity of MnJ. OVA-specific IgG1 (**a**) and total IgG (**b**) from the WT, *Sting1*^−⁄−^, *Pycard*^−⁄−^, *Nlrp3*^−⁄−^, *Sting1*^−⁄−^*Pycard*^−⁄−^, and *Sting1*^−⁄−^*Nlrp3*^−⁄−^ mice were quantified by ELISA on day 14 after intramuscular immunization with OVA (10 μg) or OVA (10 μg) + MnJ (10 μg) on days 0 and 7 (*n* > 5). **c** OVA-specific cytotoxicity was measured on day 21 in an in vivo killing assay (mice were immunized on days 0, 7, and 14, *n* = 5). **d** OVA-specific IgA from the WT, *Sting1*^−⁄−^, *Pycard*^−⁄−^, and *Sting1*^−⁄−^*Pycard*^−⁄−^ DKO mice was quantified by ELISA on day 21 after intranasal immunization with OVA (10 μg) or OVA (10 μg) + MnJ (5 μg) on days 0, 7, and 14 (*n* = 6). **e** CD45.1^+^ OT-II CD4^+^ T cells were labeled with CFSE and transferred to CD45.2^+^ WT, *Sting1*^−⁄−^, *Pycard*^−⁄−^, and *Sting1*^−⁄−^*Pycard*^−⁄−^ DKO mice. These mice were then immunized with PBS, OVA (1 μg), or OVA (1 μg) + MnJ (10 μg). After 3 days, T-cell proliferation was analyzed by FACS (*n* = 3). **f** WT, *Sting1*^−⁄−^, *Pycard*^−⁄−^, and *Sting1*^−⁄−^*Pycard*^−⁄−^ DKO mice were immunized as in (**a**). Splenocytes were collected on day 21 and stimulated with OVA peptides. IFNγ and IL-2 secretion by T cells was measured by ELISA (*n* = 3). **g** WT mice were immunized with MnJ (100 μg) or LPS (20 μg) + MnJ (100 μg) intramuscularly for the indicated times. Lysates of draining lymph node cells were analyzed by immunoblotting with the indicated antibodies. **h** WT mice, WT mice treated with anakinra (intraperitoneally, 10 mg/kg/d) or etanercept (intraperitoneally, 2 mg/kg/d), *Pycard*^−⁄−^ mice, and *Il18*^−⁄−^ mice were immunized with OVA (10 μg) + MnJ (50 μg) intraperitoneally on days 0 and 7, and OVA-specific and total IgG levels were measured by ELISA on day 14 (*n* = 5). **i** WT mice were injected intraperitoneally with PBS, Alhydrogel^®^ adjuvant (20 mg/kg), MnJ (5 mg/kg), or LPS (20 mg/kg). After 24 h, IL-1β and IL-18 levels in peritoneal lavage fluids or sera were measured by ELISA (*n* = 5). **j** Macrophages and neutrophils in peritoneal lavage fluids from mice (**i**) were analyzed by FACS (*n* = 3). One representative experiment of at least three independent experiments is shown, and each was performed in triplicate. Error bars represent the SEM; data were analyzed by an unpaired *t*-test. ns not significant; *P < 0.05; **P < 0.01; ***P < 0.001; ****P < 0.0001

Next, *Sting1*^−⁄−^, *Mavs*^−⁄−^, *Nlrp3*^−⁄−^, *Nlrc4*^−⁄−^, *Aim2*^−⁄−^, and *Pycard*^−⁄−^ mice were used to test which pathway is important for MnJ adjuvant activity. Although *Sting1*^−⁄−^, *Pycard*^−⁄−^, and *Nlrp3*^−⁄−^ mice produced diminished OVA-specific antibody levels (Supplementary Fig. 7a), *Sting1*^−⁄−^*Pycard*^−⁄−^ (STING-ASC double knockout, DKO) mice generated extremely diminished OVA-specific antibody levels (Fig. 5a–d), suggesting that both cGAS-STING and ASC inflammasomes contributed to the adjuvant effect of MnJ. However, the cGAS-STING pathway seems more important for CTL responses (Fig. 5c). Furthermore, *Sting1*^−⁄−^*Nlrp3*^−⁄−^ mice generated slightly more antibodies than *Sting1*^−⁄−^*Pycard*^−⁄−^ mice (Fig. 5a, b), indicating the involvement of other ASC-dependent, NLRP3-independent inflammasome activation by MnJ. The distribution of immune cells was the same in the spleens and LNs of wild type (WT), *Sting1*^−⁄−^*Pycard*^−⁄−^, and *Sting1*^−⁄−^*Nlrp3*^−⁄−^ mice (Supplementary Fig. 7b). We also analyzed the expression of *Dock2*, a central regulator in Rac-dependent actin polymerization and cytoskeletal reorganization, which has been reported to be downregulated in *Pycard*^−⁄−^ but not *Nlrp3*^−⁄−^ or *Caspase1*^−⁄−^ mice.^44^ Quantitative PCR analysis revealed a similar expression of *Dock2* mRNA in WT, *Sting1*^−⁄−^, *Pycard*^−⁄−^, *Nlrp3*^−⁄−^, *Sting1*^−⁄−^*Pycard*^−⁄−^, and *Sting1*^−⁄−^*Nlrp3*^−⁄−^ mice (Supplementary Fig. 7c). Consistent with our previous observations, MnJ-promoted GC formation was impaired in *Sting1*^−⁄−^*Pycard*^−⁄−^ DKO mice (Supplementary Fig. 7d). In addition, MnJ-induced OT-II CD4^+^ T-cell proliferation disappeared in DKO mice (Fig. 5e), and IFNγ and IL-2 production by peptide-stimulated splenic T cells was sharply reduced (Fig. 5f). Again, no IL-1β induction was detected in lymph nodes from MnJ-treated mice in vivo, despite ISG production and GSDMD cleavage (Fig. 5g). OVA-specific antibody production was not impaired in *Il18*^−⁄−^ mice or WT mice treated with anakinra (IL-1 receptor antagonist) or etanercept (TNFα inhibitor) (Fig. 5h), suggesting that ASC-mediated inflammasome activation might contribute to MnJ adjuvant activity by inducing the release of other DAMPs, such as uric acid,^45^ but not by inducing the release of IL-1/18 or TNFα, as reported previously.^18,46^ Additionally, peritoneal injection of Alhydrogel® adjuvant or MnJ did not induce IL-1β or IL-18 levels in peritoneal lavage fluids or sera (Fig. 5i) but significantly stimulated the recruitment of macrophages and neutrophils in the abdominal cavity (Fig. 5j). However, CFA- or monophoryl lipid A-induced antibody production and T-cell activation did not change much among these mice (Supplementary Fig. 7e, f), probably because LPS and lipid A are very potent TLR4 activators, thus overcoming the involvement of cGAS-STING in immunization.

### MnJ is a potent adjuvant for antiviral and antitumor vaccines

**Fig. 6:**
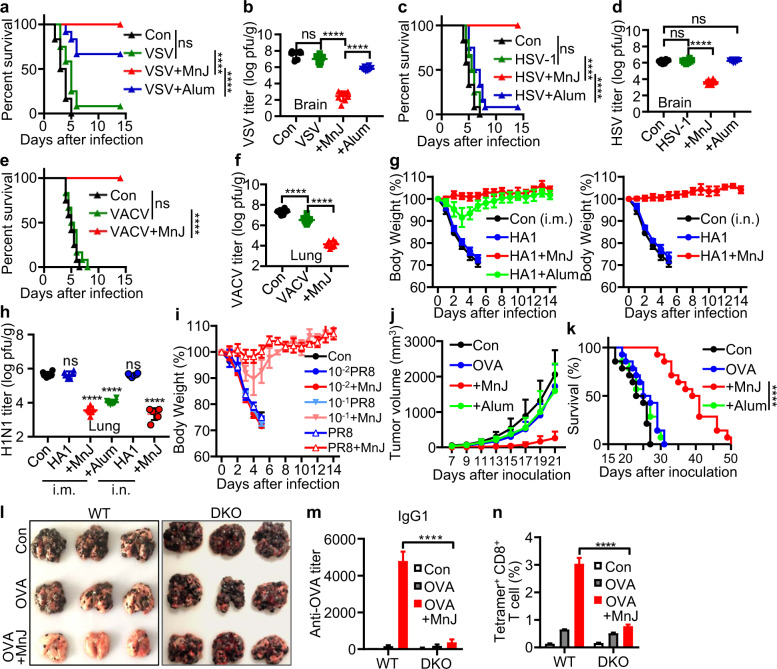
MnJ enhances the protective efficacy of inactivated viruses and subunit vaccines. **a**, **b** WT mice were immunized intramuscularly with PBS, inactivated VSV (10^5^ pfu), inactivated VSV (10^5^ pfu) + MnJ (10 μg), or inactivated VSV (10^5^ pfu) + Imject Alum (1320 μg) on day 0. On day 10, these mice were infected intravenously with a lethal dose of VSV. Their survival was monitored for 2 weeks (*n* = 12) (**a**). Viral loads in the brain were measured 5 days after infection (*n* = 8) (**b**). **c**, **d** WT mice were immunized intramuscularly with PBS, inactivated HSV-1 (10^3^ pfu), inactivated HSV-1 (10^3^ pfu) + MnJ (10 μg), or inactivated HSV-1 (10^3^ pfu) + Imject Alum (1320 μg) on day 0. On day 10, these mice were infected intraperitoneally with a lethal dose of HSV-1. The survival was monitored for 2 weeks (*n* = 12) (**c**). Viral loads in the brain were measured 5 days after infection (*n* = 8) (**d**). **e**, **f** WT mice were immunized intranasally with PBS, inactivated VACV (2 × 10^4^ pfu) or inactivated VACV (2 × 10^4^ pfu) + MnJ (5 μg) on days 0 and 7. On day 14, these mice were infected intranasally with a lethal dose of VACV. Their survival was monitored for 2 weeks (*n* = 12) (**e**). Viral loads in the lung were measured 5 days after infection (*n* = 8) (**f**). **g** The WT mice were immunized intramuscularly (i.m.) with HA1 (5 μg), HA1 (5 μg) + MnJ (10 μg), HA1 (5 μg) + Imject Alum (1320 μg) or intranasally (i.n.) with HA1 (5 μg) or HA1 (5 μg) + MnJ (10 μg) on days 0, 7, and 14. On day 21, these mice were infected intranasally with a lethal dose of PR8. Their survival was monitored for 2 weeks (*n* = 10). **h** Viral loads in lungs from mice in (**g**) were measured 5 days after infection (*n* = 6). **i** WT mice were immunized intranasally with PBS, inactivated PR8 (5 × 10^6^ pfu), 10^−1^ PR8 (5 × 10^5^ pfu) or 10^−2^ PR8 (5 × 10^4^ pfu) with or without MnJ (5 μg) on days 0 and 7. On day 14, these mice were infected intranasally with a lethal dose of PR8. Their body weight was recorded for 2 weeks (*n* = 3). **j**, **k** WT mice were immunized intramuscularly with PBS, OVA (10 μg), OVA (10 μg) + MnJ (20 μg), or OVA (10 μg) + Imject Alum (1320 μg) on days 0, 7, and 14. On day 21, these mice were inoculated with B16-OVA-Fluc cells (3 × 10^5^) subcutaneously. Tumor volume (**j**) was measured, and survival (**k**) was monitored (*n* = 14). **l**–**n** WT and DKO mice were immunized intramuscularly with PBS, OVA (10 μg), or OVA (10 μg) + MnJ (20 μg) on days 0, 7, and 14. On day 21, these mice were inoculated with B16-F10-OVA (3 × 10^5^) intravenously. Images of lung tissues were recorded 20 days after inoculation (*n* = 6) (**l**). OVA-specific IgG1 in serum was quantified by ELISA on day 20 (*n* = 6) (**m**). The percentage of tetramer^+^ CD8^+^ T cells in the spleens of these mice was analyzed by FACS on day 21 (*n* = 3) (**n**). One representative experiment of at least three independent experiments is shown, and each was performed in triplicate. Error bars represent the SEM; **b**, **d**, **f**, **h**, **m**, **n** data were analyzed by an unpaired *t*-test; **a**, **c**, **e**, **k** survival plot data were analyzed with log-rank (Mantel–Cox) tests. ns not significant; *P < 0.05; **P < 0.01; ***P < 0.001; ****P < 0.0001

Next, we tested the protective effect of MnJ-adjuvanted vaccines against various viruses. Formaldehyde-inactivated vesicular stomatitis virus (VSV), herpes simplex virus 1 (HSV-1), and vaccinia virus (VACV) were used as vaccines. The optimal dose for each inactivated virus was first determined (VSV and HSV i.m.; VACV i.n.) (Supplementary Fig. 8a–f). We found that MnJ greatly enhanced the protection efficacy of inactivated virus by ~100 times. Consistently, virus titers were decreased by at least 10^3^ times in MnJ-immunized mice (Fig. 6a–f), indicating that MnJ can be applied to virus vaccines through either intramuscular or intranasal immunization, with greatly reduced amounts of inactivated virus needed for adequate protection.

We particularly tested MnJ in influenza vaccines. Mice immunized with the virus protein HA1 (PR8) plus MnJ (i.m. or i.n.) were completely protected from lethal challenge by the H1N1 A/PR8/34 virus, while the aluminum-containing adjuvant only showed mild protection (Fig. 6g, h and Supplementary Fig. 8g–i). Importantly, MnJ enhanced the protective effect of the inactivated PR8 vaccine even when ten times less inactivated virus was used (Fig. 6i). Moreover, MnJ exhibited superior protection against heterologous influenza viruses in mice immunized with MnJ-adjuvanted inactivated PR8 or HA1 protein, followed by lethal H1N1-WSN or H3N2 challenge (Supplementary Fig. 8j).

Finally, the adjuvant effect of MnJ on cancer vaccines was evaluated. Mice were immunized three times before subcutaneous inoculation with B16-OVA melanoma cells. Tumor growth was greatly suppressed in OVA-MnJ-immunized but not OVA-aluminum-immunized mice (Fig. 6j and Supplementary Fig. 8k), in line with prominently improved survival (Fig. 6k) and increased levels of tumor-infiltrating CD4^+^ and CD8^+^ T cells (Supplementary Fig. 8l, m), confirming the CTL-inducing activity of MnJ. In a pulmonary metastasis model, OVA-MnJ immunization greatly blocked lung metastases in WT but not *Sting1*^−⁄−^*Pycard*^−⁄−^ mice (Fig. 6l and Supplementary Fig. 8n, o), which is consistent with the proliferation of CD8^+^ OT-I T cells (Supplementary Fig. 8p). In addition, OVA-specific antibody production and CD8^+^ T-cell activation analyzed by tetramer assays were detected only in MnJ-OVA-immunized WT mice (Fig. 6m, n and Supplementary Fig. 8q), suggesting systematic activation of the antitumor adaptive immune response as well as its potential in tumor therapies.

## Discussion

It may not be surprising that Mn and cGAS-STING are involved in regulating adaptive immunity, given that the cGAS-STING pathway is important in the immunosurveillance of all aberrant cells, including infected, damaged, senesced, mutated, or dead cells,^47^ and that Mn^2+^ greatly sensitizes cGAS-STING^24^ and/or directly activates cGAS.^25,26^ Furthermore, it has been well established that type I IFNs bridge innate and adaptive immunity by promoting the maturation and activation of DCs for antigen presentation; stimulating TH1 responses, especially CTL activation; and enhancing the survival of memory T cells.^48–50^ Accordingly, recent works have reported that various antitumor therapies depend on the activation of the cGAS-STING pathway^51–53^ by promoting tumor-specific antigen presentation and CTL activation.^54,55^ Consistently, we recently reported that Mn^2+^ is also essential in innate immune surveillance and supports adaptive immune responses against tumors, as Mn-deficient mice had significantly enhanced tumor growth and metastasis, with greatly reduced levels of tumor-infiltrating CD8^+^ T and NK cells.^56^ Mechanistically, we provided evidence to show that Mn^2+^ supported immune responses by (1) promoting the differentiation of Mo-DCs; (2) promoting DC maturation and antigen presentation; (3) inducing the levels of chemokines for immune cell homing; (4) facilitating antigen uptake; and (5) promoting GC formation for better antibody selection and production.

Interestingly, Mn^2+^ induced APCs to produce both IFNβ and various IFNαs, whereas LPS induced only IFNβ. This unique feature may account for the superior adjuvant activities of MnJ presented in this work, as well as that the production of several good antibodies against proteins with low immunogenicity, which are very difficult to generate antibody by other adjuvants, has been successively induced by MnJ in different laboratories (unpublished data and personal communications). Moreover, Mn^2+^ did not induce the production of the proinflammatory cytokines IL-1 and IL-18 in humans or mice, even though they stimulated similar production of IL-6 and TNFα. Although both cGAS-STING and TLR4 converge to activate transcription factors, including IRF3 and NF-κB, which lead to the production of cytokines, including type I IFNs, there are obvious differences between these two pathways. Previous work demonstrated that cGAS-STING utilizes a unique NF-κB-activating cascade through the TRIM32/56-NEMO-IKKβ axis.^57–59^ Moreover, whereas both IRF3 and IRF7 are required for TLR4 activation, solely IRF3 is required for cGAS-STING via a distinct activating mechanism.^17,57,60^ We thus speculated that the different expression levels of *Il1β* and *Il18* might result from the different activation of the NF-κB pathway. In addition, the availability of different adapter proteins or even transcriptional factor subunits within different cells may dictate the induction of altered sets of cytokines.^61^ The detailed mechanisms are worthy of further investigation.

The role of the NLRP3 inflammasome in aluminum-containing adjuvants is still controversial,^8^ which may be attributed to the use of different aluminum salts or mouse backgrounds. It has also been proposed that ASC has an inflammasome-independent role in shaping adaptive immunity by regulating the expression of Dock2 for antigen uptake and lymphocyte mobility.^44^ However, we found that STING, NLRP3, or ASC deficiency did not affect the expression of Dock2 in macrophages or lymphocytes. Our results suggested that Mn^2+^ regulates adaptive immunity partly in an inflammasome-dependent but inflammatory cytokine-independent manner, in which some factors might be upregulated by Mn^2+^ and released by pyroptotic death of cells. Nevertheless, even though Mn^2+^ treatment alone on macrophages or PBMCs did not induce IL-1/18 production, these cells may produce functional IL-1/18 when treated with inactivated pathogens (the first signal for inflammasome activation) plus Mn^2+^ (the second signal).^62^

MnJ has several unique features that indicate that it is a promising adjuvant. The good adjuvant activity of MnJ is probably due to (1) its ability to activate both the cGAS-STING pathway and NLRP3 inflammasome; (2) its ability to induce the production of various IFN-αs, leading to a distinguishable APC differentiation and maturation pattern; and (3) its ability to act as both an immune activator and a delivery system, inducing humoral, cellular, and mucosal immune responses without obviously detected side effects. Because of these benefits and its stability against repeated freeze-thaw treatment, Mn-based adjuvants would be especially useful in veterinary vaccines, with the following three additional advantages: (1) MnJ showed good dose-dependent adjuvant activity i.m. and i.n.; (2) a high MnJ dose (up to several mg/injection) and repeated administration to mice, rabbits, or pigs (data not shown) did not cause visible damage or inflammation; and (3) mammals maintained tissue Mn levels via tight control of both absorption and excretion, as normally only 1–5% of ingested Mn is absorbed into the body,^63^ and excessive dietary Mn causes reduced Mn absorption and enhanced Mn metabolism and excretion.^64–66^ Therefore, even highly elevated Mn levels in meats caused by Mn-containing veterinary vaccines (which may never occur) would not likely increase gastrointestinal Mn absorption by consumers. In fact, Mn contents in whole grains, rice, and nuts are ~30 mg Mn/kg or more or even 110–140 mg Mn/kg in wheat bran, which are much higher than those in mammals (between 0.3 and 2.9 mg Mn/kg wet tissue weight), confirming the tight Mn absorption regulation by animals.

Mn-based chemical compounds have long been widely used in the clinic. For example, manganese dipyridoxal diphosphate is used to estimate the functionality of hepatocytes for good magnetic resonance contrast and quick clearance.^67^ In particular, MnO_2_ nanoparticles were shown to have beneficial effects in antitumor therapies due to their biocompatibility, tumor hypoxia modulation, and ROS reduction.^68,69^ A hollow MnO_2_ nanodrug delivery system was able to enhance antitumor effects, with a primary distribution in tumors.^70^ These clinical results may also be nicely attributed to the adjuvant activity or immunostimulatory effect of Mn^2+^ released from these compounds. Importantly, this work demonstrated that MnJ immunization stimulated the differentiation of tumor antigen-specific CTLs, indicating great potential for cancer vaccines. In addition, the component simplicity and steadiness of MnJ and the low cost and wide availability of Mn make this adjuvant even more promising.

## Materials and methods

### Human subjects

This study was approved by the Ethical Committee on Human Research of Peking University and was in accordance with the Declaration of Helsinki. PBMCs were isolated from the peripheral blood of volunteers (Supplementary Table 2) using Histopaque-1077 (Sigma, 10771) through consecutive centrifugation. PBMCs were seeded in 12-well plates at a final concentration of 5 × 10^6^ cells/well with Opti-MEM (Gibco) and treated with the indicated concentrations of LPS or Mn^2+^.

### Mice

WT C57BL/6-specific pathogen-free (SPF) mice were purchased from Vital River, China. The *Sting1*^−⁄−^ and *Cgas*^−⁄−^ mice have been previously described.^24,56^ The OT-I (C57BL/6-Tg (TcraTcrb) 1100 Mjb/J) mice and OT-II (B6. Cg-Tg (TcraTcrb) 425Cbn/J) mice were gifts from Dr. Yan Shi. The *Mavs*^−⁄−^ mice were gifts from Dr. Zhijian Chen. The *Irf3*^−⁄−^*Irf7*^−⁄−^ mice were gifts from Dr. Tadatsugu Taniguchi. The *Ifnar1*^−⁄−^ and *Il18*^−⁄−^ mice were gifts from Dr. Rongbin Zhou. The *Pycard*^−⁄−^ mice, *Aim2*^−⁄−^ mice, *Nlrp3*^−⁄−^ mice, and *Nlrc4*^−⁄−^ mice were gifts from Dr. Vishva M. Dixit. The *Sting1*^−⁄−^*Pycard*^−⁄−^ DKO mice were generated by crossing *Sting1*^−⁄−^ mice and *Pycard*^−⁄−^ mice. The *Sting1*^−⁄−^*Nlrp3*^−⁄−^ mice were generated by crossing *Sting1*^−⁄−^ mice and *Nlrp3*^−⁄−^ mice.

All mice were bred and kept under SPF conditions in the Laboratory Animal Center of Peking University in accordance with the National Institute of Health Guide for Care and Use of Laboratory Animals.

### Cell lines

L929-ISRE, MDCK, BHK21, B16-F10-OVA, B16-F10-OVA-GFP cells were cultured in DMEM (Gibco) supplemented with 10% FBS (Gibco), 5 μg/ml penicillin, and 10 μg/ml streptomycin. THP1 and THP1-derived knockout cells were cultured in RPMI-1640 (Gibco) medium supplemented with 10% FBS (Gibco). THP1 gene-specific knockout cells were generated by the CRISPR-cas9 system with the gRNA sequence listed in Supplementary Table 3.

Peritoneal macrophages were harvested from mice 6 days after thioglycollate (BD, Sparks, MD) injection and cultured in DMEM supplemented with 5% FBS.

For induction of immature BMDCs, bone marrow cells were isolated from WT mice and the indicated knockout mice and cultured in RPMI-1640 (Gibco) medium supplemented with 10% FBS (Gibco), 20 ng/ml mIL-4 (Genscript), and 20 ng/ml mGM-CSF (Genscript) at 37 °C and with 5% CO_2_. On day 3, half of the culture supernatant was changed to fresh medium. On day 7, nonadherent cells were collected and used immediately. DC purity was confirmed with PE-labeled anti-CD11c antibodies (Biolegend, Cat# 117307) by FACS.

For induction of human Mo-DCs, monocytes were isolated from human PBMCs with a MojoSort™ Human Pan Monocyte Isolation Kit (Biolegend, Cat# 480060) and cultured in RPMI-1640 (Gibco) medium supplemented with 10% FBS (Gibco), 100 ng/ml hIL-4 (Genscript), and 100 ng/ml hGM-CSF (Genscript) at 37 °C and with 5% CO_2_. On day 3, half of the culture supernatant was changed to fresh medium. On day 7, nonadherent cells were collected and used immediately. DC purity was confirmed with APC-labeled anti-CD11c antibodies (Biolegend, Cat# 301613) by FACS.

### Virus infection

The influenza virus A/WSN/1933 (H1N1) strain was obtained from Wenjun Liu, Institute of Microbiology, CAS, China. The A/PR8/34 (H1N1) strain was obtained from Yonghui Zhang, Tsinghua University. VACV (Western Reserve strain), H3N2 subtype A/Jiangxi/2005, and H9N2 subtype A/Chicken/Liaoning/1/00 were obtained from Min Fang, Institute of Microbiology, CAS. HSV-1 (WT F strain) and VSV (Indiana strain) were obtained from Hongbing Shu, Wuhan University.

For mouse survival experiments, 6–8-week-old mice were infected with lethal titers of VSV (5 × 10^8^ pfu per mouse) intravenously, HSV-1 (1 × 10^7^ pfu per mouse) intraperitoneally, VACV (1 × 10^7^ pfu per mouse) i.n., H1N1-PR8 (1 × 10^6^ pfu per mouse) i.n., H1N1-WSN (1 × 10^6^ pfu per mouse) i.n., and H3N2 (1 × 10^6^ pfu per mouse) i.n. after immunization.

For plaque assays, VSV, HSV-1, and VACV titers were measured in BHK21 cells, and influenza virus titers were measured in MDCK cells. BHK21 cell monolayers (100% confluence in 24-well plates) were washed with phosphate-buffered NS (PBS) and infected with different dilutions of virus for 1 h at 37 °C. Then, virus inoculums were removed and washed twice with PBS. Cell monolayers were overlaid with 0.5% methylcellulose in FBS-free DMEM. Forty-eight to 72 h later, the cells were fixed with 0.5% glutaraldehyde and stained with 1% crystal violet dissolved in 70% ethanol. MDCK cell monolayers (100% confluence in 12-well plates) were infected as the BHK21 cells were, overlaid with agar overlay medium (DMEM containing 1% low-melting-point agarose and 1 μg/ml TPCK-treated trypsin) and incubated at 37 °C for 48–72 h. Plaques were counted to determine virus titers.

For inactivated virus preparation, different viruses were purified by adding 4% PEG-6000 and 2% NaCl to settle overnight at 4 °C. Then, viruses were centrifuged at 8000 × *g* for 2 h, and precipitates were dissolved in PBS. The purified viruses were inactivated by adding 0.1% formaldehyde and rotated overnight at 37 °C. After that, viruses were confirmed to be completely inactivated by a plaque assay.

### Clinical scoring of IAV model

The scoring system was based on coat condition, posture, and activity according to previously described methods:^71^ ruffled fur (absent 0, mild 1, and severe 2), hunched back (absent 0, mild 1, and severe 2), and activity (normal 0, reduced 1, and severely reduced 2). The final score = ruffled fur + hunched back + activity.

### Generation and purification of IAVs

A/PR8/34 (H1N1), A/WSN/1933 (H1N1), A/Jiangxi/262/2005 (H3N2), and H9N2 subtype A/Chicken/Liaoning/1/00 viruses were generated in 10-day-old embryonated chicken eggs for 2 days at 37 °C. The allantoic fluids were collected. Then, the influenza viruses were purified by sucrose density gradient centrifugation at 20,000 × *g* for 2 h at 4 °C. Finally, viruses were dissolved in PBS, and titers were determined by a plaque assay.

### Type I IFN bioassay

The type I IFN concentration was measured as previously described.^72^ Briefly, an IFN-sensitive luciferase vector was constructed by cloning an IFN-stimulated response element into the pGL3-Basic vector (Promega) and was stably transfected into L929 and HT1080 cells. L929-ISRE or HT1080-ISRE^73^ cells were seeded into 96-well plates and incubated with mouse or human cell culture supernatants. Recombinant mouse or human IFNβ (R&D Systems) was used as a standard. Four hours later, the cells were lysed and measured by a Luciferase Reporter Assay System (Promega).

### Quantitative reverse transcription PCR analysis

Total RNA was isolated using TRIzol reagent (Invitrogen) according to the manufacturer’s instructions. 1 μg of total RNA was converted into cDNA with an oligo dT primer and RevertAid reverse transcriptase (Thermo Scientific). PCR was performed with gene-specific primer sets (Supplementary Table 4). Quantitative real-time PCR was performed with SYBR green incorporation on a LightCycler® 96 System (Roche), and the data are presented as the mRNA accumulation index (2^−△△Ct^).

### Protein expression and purification

The HA1 (11–324 aa) plasmid was obtained from Dr. Yonghui Zhang. The S1 subunit plasmid of SARS-CoV-2 was obtained from Drs. Lu Lu and Peihui Wang. The recombinant HA1 and S1 were expressed in *E. coli* BL21 (DE3) with 1 mM IPTG induction for 5 h at 37 °C. The proteins were purified according to published procedures.^74^

### Mouse immunization

Mice were immunized with a prime-boost strategy. For intramuscular immunization, each mouse was immunized with 10 μg of OVA (InvivoGen, Cat# vac-pova) alone or with MnJ, Imject® Alum (Thermo), Alhydrogel® adjuvant 2% (InvivoGen), Adju-Phos® adjuvant (InvivoGen), Freund’s Complete Adjuvant (Sigma), Freund’s Incomplete Adjuvant (Sigma), Sigma Adjuvant System (Sigma), polyethylenimine (Polysciences), or MF59 (gift from Dr. Yonghui Zhang) suspended in PBS or normal saline with a final volume of 100 μl. Then, the mice were boosted on days 7 and 14. For intranasal immunization, each mouse was immunized with 10 μg of OVA alone or with MnJ, cholera toxin B subunit (Sigma) suspended in PBS or normal saline with a final volume of 20 μl after anesthetization. Then, the mice were boosted on days 7 and 14.

### Antibody titer test

Serum was collected from whole blood by centrifugation. Bronchoalveolar lavage fluid was collected with 500 μl of PBS from the lung. Oral lavage fluid was collected with 200 μl of PBS from the oral cavity. ELISA plates were coated with 2 μg/ml antigens (OVA, HA1, NP-BSA, EV71, HBsAg, HBSS1, or SARS-CoV-2-S1) in PBS at 4 °C overnight. After washing five times with 200 μl of Wash Buffer (0.05% Tween 20 in PBS), plates were blocked with 200 μl of Block Buffer (2% BSA in PBS) for 2 h at 37 °C. After washing five times, 100 μl of diluted samples was added to plates and incubated for 2 h at 37 °C. After washing five times, the plates were incubated with HRP-labeled antibodies for 2 h at 37 °C. After washing five times, the plates were incubated with 100 μl of 1 × TMB Solution (eBioscience) for 30 min at 37 °C, followed by the addition of 100 μl of Stop Solution (MultiSciences). The optical density (OD) was read at 450 and 570 nm by a FlexStation 3 (Molecular Devices). Readings at 570 nm were subtracted from the readings at 450 nm. Antibody titers were obtained by plotting the maximum serum dilution that gave an OD > 2 × background.

### In vitro BMDC stimulation

Immature BMDCs (1 × 10^6^) were plated onto 12-well plates and then treated with the indicated concentrations of MnCl_2_ or LPS for 20 h. The BMDCs were stained with anti-CD86, anti-CD80, and anti-CD40 antibodies (Biolegend). The cell-surface costimulatory molecules CD86, CD80, and CD40 were analyzed by FACS.

### In vivo T-cell proliferation

OT-I CD8^+^ T cells or OT-II CD4^+^ T cells were isolated from CD45.1^+^ OT-I or OT-II mice by a Mouse CD8 or CD4 T Cell Isolation Kit (Biolegend). CFSE (1 μM)-labeled CD45.1^+^ OT-I CD8^+^ or OT-II CD4^+^ T cells (2 × 10^6^) were transferred intravenously into naive CD45.2^+^ mice before immunization the next day. Inguinal lymph nodes of the vaccinated mice were obtained 3 days after immunization, and separated CD4^+^ CD45.1^+^ or CD8^+^ CD45.1^+^ T cells were analyzed by FACS using Precision Count Beads (Biolegend, Cat# 424902).

### In vivo antigen uptake and Mo-DC accumulation analysis

C57BL/6 mice were vaccinated in both inguinal regions subcutaneously with PBS, PE, PE + MnJ, or PE + aluminum. Inguinal dLNs were collected 12 or 24 h after immunization. dLN cells were labeled with DAPI and anti-CD11c (Biolegend, Cat# 117325), anti-F4/80 (Biolegend, Cat# 123115), anti-CD8a (Biolegend, Cat# 100721), and anti-CD64 antibodies (Biolegend, Cat# 139315) to identify APCs. The ratio of PE^+^ APCs (CD11c^+^ or F4/80^+^ cells) from dLN cells and the ratio of Mo-DCs (CD64^+^ CD8a^+^) in APCs (CD11c^+^ or F4/80^+^ cells) were analyzed by FACS.

### In vivo cytotoxicity assay

C57BL/6 mice were vaccinated with OVA (10 μg), OVA (10 μg) + MnJ (20 μg), or OVA (10 μg) + aluminum (1320 μg) on days 0, 7, and 14. On day 21, vaccinated animals were intravenously injected with 2 × 10^6^ donor splenocytes from naive C57BL/6 mice. Half were labeled with 0.5 μM CFSE (Biolegend, Cat# 423801), and the other half were labeled with 5 μM CFSE for 10 min at 37 °C. The cells stained at high CFSE concentrations were pulsed with 10 μg/ml SIINFEKL peptide for 90 min at 37 °C.^75^ Thirty-six hours after transfer, splenocytes were collected, and specific killing was defined as the percentage of specific lysis = (transferred ratio − experimental ratio) × 100%.

### Germinal center analysis

Inguinal dLNs were collected 7 days after immunization. Single LN cells were stained with FITC-labeled anti-CD3 (Biolegend, Cat# 100203), APC-labeled anti-CD4 (Biolegend, Cat# 100411), PE-labeled anti-PD1 (Biolegend, Cat# 135205), and PECy7-labeled anti-CXCR5 antibodies (Biolegend, Cat# 145515) to analyze Tfh cells or Alexa Fluor® 700-labeled anti-CD45 (Biolegend, Cat# 103127), FITC-labeled anti-B220 (Biolegend, Cat# 103205), APC-labeled anti-GL7 (Biolegend, Cat# 144617), and PE-labeled anti-Fas antibodies (Biolegend, Cat# 152607) to analyze GC B cells.

### Tumor model

For subcutaneous tumors, C57BL/6 mice were vaccinated with OVA (10 μg), OVA (10 μg) + MnJ (20 μg), or OVA (10 μg) + aluminum (1320 μg) on days 0, 7, and 14. On day 21, vaccinated animals were inoculated subcutaneously in the right hind flank with 3 × 10^5^ B16-F10-OVA cells. Tumor sizes were measured every 2 days with electronic calipers and calculated by length (mm) × width (mm) × width (mm)/2. Mice with tumors larger than 20 mm on the longest axis were euthanized. IVIS images were captured with a Caliper IVIS Lumina II (Caliper Life Sciences) instrument following light anesthesia with pentobarbital sodium and intraperitoneal injection of D-luciferin (Cayman Chemical, 14681) (0.5 mg/g per mouse). Images were quantified using Living Image 4.0.

For metastatic tumors, C57BL/6 mice were vaccinated with OVA (10 μg) or OVA (10 μg) + MnJ (20 μg) on days 0, 7, and 14. On day 21, vaccinated animals were inoculated intravenously with 3 × 10^5^ B16-F10-OVA-GFP cells. Mice were euthanized 20 days after inoculation. Images of lungs and HE-stained lung sections were recorded.

### Quantification and statistical analysis

All results are expressed as the mean ± SEM. Unpaired two-tailed *t*-tests were used for comparison of two groups. Two-way ANOVA was performed when both time and treatment were compared. For the survival studies, a log-rank (Mantel–Cox) test was used. *P* < 0.05 was considered statistically significant and denoted as follows: *P < 0.05, **P < 0.01, ***P < 0.001, and ****P < 0.0001. Statistical analyses were performed by using GraphPad Prism.

## Supplementary information

SUPPLEMENTAL MATERIAL

## Data Availability

Data from the RNA-seq analysis are available through the GEO database (Accession Number GSE126586 (Fig. 1a)).

## References

[1] Petrovsky N, Aguilar JC (2004). Vaccine adjuvants: current state and future trends. Immunol. Cell Biol..

[2] Apostolico Jde S, Lunardelli VA, Coirada FC, Boscardin SB, Rosa DS (2016). Adjuvants: classification, modus operandi, and licensing. J. Immunol. Res..

[3] Pashine A, Valiante NM, Ulmer JB (2005). Targeting the innate immune response with improved vaccine adjuvants. Nat. Med..

[4] Kaurav M, Madan J, Sudheesh MS, Pandey RS (2018). Combined adjuvant-delivery system for new generation vaccine antigens: alliance has its own advantage. Artif. Cells Nanomed. Biotechnol..

[5] McKee AS, Marrack P (2017). Old and new adjuvants. Curr. Opin. Immunol..

[6] Clements CJ, Griffiths E (2002). The global impact of vaccines containing aluminium adjuvants. Vaccine.

[7] O’Hagan DT, Friedland LR, Hanon E, Didierlaurent AM (2017). Towards an evidence based approach for the development of adjuvanted vaccines. Curr. Opin. Immunol..

[8] Marrack P, McKee AS, Munks MW (2009). Towards an understanding of the adjuvant action of aluminium. Nat. Rev. Immunol..

[9] Barber GN (2015). STING: infection, inflammation and cancer. Nat. Rev. Immunol..

[10] Luo M (2017). A STING-activating nanovaccine for cancer immunotherapy. Nat. Nanotechnol..

[11] Corrales L (2015). Direct activation of STING in the tumor microenvironment leads to potent and systemic tumor regression and immunity. Cell Rep..

[12] Del Giudice G, Rappuoli R, Didierlaurent AM (2018). Correlates of adjuvanticity: a review on adjuvants in licensed vaccines. Semin. Immunol..

[13] Sun L, Wu J, Du F, Chen X, Chen ZJ (2013). Cyclic GMP-AMP synthase is a cytosolic DNA sensor that activates the type I interferon pathway. Science.

[14] Ishikawa H, Barber GN (2008). STING is an endoplasmic reticulum adaptor that facilitates innate immune signalling. Nature.

[15] Zhong B (2008). The adaptor protein MITA links virus-sensing receptors to IRF3 transcription factor activation. Immunity.

[16] Sun W (2009). ERIS, an endoplasmic reticulum IFN stimulator, activates innate immune signaling through dimerization. Proc. Natl Acad. Sci. USA.

[17] Tang CK (2013). The chemotherapeutic agent DMXAA as a unique IRF3-dependent type-2 vaccine adjuvant. PLoS ONE.

[18] Blaauboer SM, Gabrielle VD, Jin L (2014). MPYS/STING-mediated TNF-alpha, not type I IFN, is essential for the mucosal adjuvant activity of (3’-5’)-cyclic-di-guanosine-monophosphate in vivo. J. Immunol..

[19] Li XD (2013). Pivotal roles of cGAS-cGAMP signaling in antiviral defense and immune adjuvant effects. Science.

[20] Carroll EC (2016). The vaccine adjuvant chitosan promotes cellular immunity via DNA sensor cGAS-STING-dependent induction of type I interferons. Immunity.

[21] Horning KJ, Caito SW, Tipps KG, Bowman AB, Aschner M (2015). Manganese is essential for neuronal health. Annu. Rev. Nutr..

[22] Kwakye GF, Paoliello MM, Mukhopadhyay S, Bowman AB, Aschner M (2015). Manganese-induced parkinsonism and Parkinson’s disease: shared and distinguishable features. Int. J. Environ. Res. Public Health.

[23] Waldron KJ, Rutherford JC, Ford D, Robinson NJ (2009). Metalloproteins and metal sensing. Nature.

[24] Wang C (2018). Manganese increases the sensitivity of the cGAS-STING pathway for double-stranded DNA and is required for the host defense against DNA viruses. Immunity.

[25] Hooy RM, Massaccesi G, Rousseau KE, Chattergoon MA, Sohn J (2020). Allosteric coupling between Mn^2+^ and dsDNA controls the catalytic efficiency and fidelity of cGAS. Nucleic Acids Res..

[26] Zhao Z (2020). Mn(2+) directly activates cGAS and structural analysis suggests Mn(2+) induces a noncanonical catalytic synthesis of 2’3’-cGAMP. Cell Rep..

[27] Deng J, Yu XQ, Wang PH (2019). Inflammasome activation and Th17 responses. Mol. Immunol..

[28] Eisenbarth SC, Colegio OR, O’Connor W, Sutterwala FS, Flavell RA (2008). Crucial role for the Nalp3 inflammasome in the immunostimulatory properties of aluminium adjuvants. Nature.

[29] Li H, Willingham SB, Ting JPY, Re F (2008). Cutting edge: inflammasome activation by alum and alum’s adjuvant effect are mediated by NLRP3. J. Immunol..

[30] Kool M (2008). Cutting edge: alum adjuvant stimulates inflammatory dendritic cells through activation of the NALP3 inflammasome. J. Immunol..

[31] Gaidt MM (2017). The DNA inflammasome in human myeloid cells is Initiated by a STING-cell death program upstream of NLRP3. Cell.

[32] Swanson KV (2017). A noncanonical function of cGAMP in inflammasome priming and activation. J. Exp. Med..

[33] Sarkar, S. et al. Manganese activates NLRP3 inflammasome signaling and propagates exosomal release of ASC in microglial cells. *Sci. Signal*. **12**10.1126/scisignal.aat9900 (2019). 10.1126/scisignal.aat9900 PMC6420319 30622196

[34] Wang D (2017). The role of NLRP3-CASP1 in inflammasome-mediated neuroinflammation and autophagy dysfunction in manganese-induced, hippocampal-dependent impairment of learning and memory ability. Autophagy.

[35] Wree A (2017). NLRP3 inflammasome driven liver injury and fibrosis: roles of IL-17 and TNF in mice. Hepatology.

[36] Amini, M. A. et al. Combining tumor microenvironment modulating nanoparticles with doxorubicin to enhance chemotherapeutic efficacy and boost antitumor immunity. *J. Natl Cancer Inst.*10.1093/jnci/djy131 (2018). 10.1093/jnci/djy131 30239773

[37] Hussain SM (2006). The interaction of manganese nanoparticles with PC-12 cells induces dopamine depletion. Toxicol. Sci..

[38] Chen H (2012). Optimisation of prime-boost immunization in mice using novel protein-based and recombinant vaccinia (Tiantan)-based HBV vaccine. PLoS ONE.

[39] Mond JJ, Kokai-Kun JF (2008). The multifunctional role of antibodies in the protective response to bacterial T cell-independent antigens. Curr. Top. Microbiol. Immunol..

[40] Hutter J, Lepenies B (2015). Carbohydrate-based vaccines: an overview. Methods Mol. Biol..

[41] Huang HR (2018). Dectin-3 recognizes glucuronoxylomannan of *Cryptococcus neoformans* serotype AD and *Cryptococcus gattii* serotype B to initiate host defense against cryptococcosis. Front. Immunol..

[42] Cioncada R (2017). Vaccine adjuvant MF59 promotes the intranodal differentiation of antigen-loaded and activated monocyte-derived dendritic cells. PLoS ONE.

[43] Langlet C (2012). CD64 expression distinguishes monocyte-derived and conventional dendritic cells and reveals their distinct role during intramuscular immunization. J. Immunol..

[44] Ippagunta SK (2011). The inflammasome adaptor ASC regulates the function of adaptive immune cells by controlling Dock2-mediated Rac activation and actin polymerization. Nat. Immunol..

[45] Kool M (2008). Alum adjuvant boosts adaptive immunity by inducing uric acid and activating inflammatory dendritic cells. J. Exp. Med..

[46] Oleszycka E (2016). IL-1alpha and inflammasome-independent IL-1beta promote neutrophil infiltration following alum vaccination. FEBS J..

[47] Ablasser, A. & Chen, Z. J. cGAS in action: expanding roles in immunity and inflammation. *Science* **363**10.1126/science.aat8657 (2019). 10.1126/science.aat8657 30846571

[48] Dunn GP (2005). A critical function for type I interferons in cancer immunoediting. Nat. Immunol..

[49] Zitvogel L, Galluzzi L, Kepp O, Smyth MJ, Kroemer G (2015). Type I interferons in anticancer immunity. Nat. Rev. Immunol..

[50] Dubensky TW, Reed SG (2010). Adjuvants for cancer vaccines. Semin Immunol..

[51] Deng L (2014). STING-dependent cytosolic DNA sensing promotes radiation-induced type I interferon-dependent antitumor immunity in immunogenic tumors. Immunity.

[52] Woo SR (2014). STING-dependent cytosolic DNA sensing mediates innate immune recognition of immunogenic tumors. Immunity.

[53] Woo SR, Corrales L, Gajewski TF (2015). The STING pathway and the T cell-inflamed tumor microenvironment. Trends Immunol..

[54] Chen Q, Sun L, Chen ZJ (2016). Regulation and function of the cGAS-STING pathway of cytosolic DNA sensing. Nat. Immunol..

[55] Wang H (2017). cGAS is essential for the antitumor effect of immune checkpoint blockade. Proc. Natl Acad. Sci. USA.

[56] Lv M (2020). Manganese is critical for antitumor immune responses via cGAS-STING and improves the efficacy of clinical immunotherapy. Cell Res.

[57] Fang R (2017). NEMO-IKKbeta are essential for IRF3 and NF-kappaB activation in the cGAS-STING pathway. J. Immunol..

[58] Ishikawa H, Ma Z, Barber GN (2009). STING regulates intracellular DNA-mediated, type I interferon-dependent innate immunity. Nature.

[59] Abe T, Barber GN (2014). Cytosolic-DNA-mediated, STING-dependent proinflammatory gene induction necessitates canonical NF-kappaB activation through TBK1. J. Virol..

[60] Hornung V (2014). SnapShot: nucleic acid immune sensors, part 1. Immunity.

[61] Fang R (2017). MAVS activates TBK1 and IKKepsilon through TRAFs in NEMO dependent and independent manner. PLoS Pathog..

[62] Jo EK, Kim JK, Shin DM, Sasakawa C (2016). Molecular mechanisms regulating NLRP3 inflammasome activation. Cell Mol. Immunol..

[63] Williams, M. et al. in *Toxicological Profile for Manganese* (Agency for Toxic Substances and Disease Registry (ATSDR) Toxicological Profiles, 2012). 24049862

[64] Davis CD, Zech L, Greger JL (1993). Manganese metabolism in rats: an improved methodology for assessing gut endogenous losses. Proc. Soc. Exp. Biol. Med..

[65] Britton AA, Cotzias GC (1966). Dependence of manganese turnover on intake. Am. J. Physiol..

[66] Mahoney JP, Small WJ (1968). Studies on manganese. 3. The biological half-life of radiomanganese in man and factors which affect this half-life. J. Clin. Investig..

[67] Young SW, Simpson BB, Ratner AV, Matkin C, Carter EA (1989). MRI measurement of hepatocyte toxicity using the new MRI contrast agent manganese dipyridoxal diphosphate, a manganese/pyridoxal 5-phosphate chelate. Magn. Reson. Med..

[68] Gordijo CR (2015). Design of hybrid MnO_2_-polymer-lipid nanoparticles with tunable oxygen generation rates and tumor accumulation for cancer treatment. Adv. Funct. Mater..

[69] Abbasi AZ (2016). Hybrid manganese dioxide nanoparticles potentiate radiation therapy by modulating tumor hypoxia. Cancer Res..

[70] Yang G (2017). Hollow MnO_2_ as a tumor-microenvironment-responsive biodegradable nano-platform for combination therapy favoring antitumor immune responses. Nat. Commun..

[71] Honda-Okubo Y, Ong CH, Petrovsky N (2015). Advax delta inulin adjuvant overcomes immune immaturity in neonatal mice thereby allowing single-dose influenza vaccine protection. Vaccine.

[72] Jiang Z (2005). CD14 is required for MyD88-independent LPS signaling. Nat. Immunol..

[73] You F (2009). PCBP2 mediates degradation of the adaptor MAVS via the HECT ubiquitin ligase AIP4. Nat. Immunol..

[74] Song L (2008). Efficacious recombinant influenza vaccines produced by high yield bacterial expression: a solution to global pandemic and seasonal needs. PLoS ONE.

[75] Kobiyama K (2014). Nonagonistic Dectin-1 ligand transforms CpG into a multitask nanoparticulate TLR9 agonist. Proc. Natl Acad. Sci. USA.

