# Supplementary material for: Manganese salts function as potent adjuvants
Source: Cell Mol Immunol. 2021 Mar 25;18(5):1222–34. doi: 10.1038/s41423-021-00669-w (PMC8093200; doi:10.1038/s41423-021-00669-w)
Supplement: Supplementary file 1 — SUPPLEMENTAL MATERIAL [file 41423_2021_669_MOESM1_ESM.pdf]

Supplementary Materials for

Manganese salts function as potent adjuvants

Rui Zhang, Chenguang Wang, Yukun Guan, Xiaoming Wei, Mengyin Sha, Mengran Yi, Miao Jing, Mengze Lv, Wen Guo, Jing Xu, Yi Wan, Xin-Ming Jia, Zhengfan Jiang*

*Corresponding author. Email: jiangzf@pku.edu.cn

The PDF file includes:

Materials and Methods

Supplementary Fig. 1. Mn^{2+} activates NLRP3 independent of cGAS or STING.

Supplementary Fig. 2. ROS, K^{+} efflux and Ca^{2+} release are required for Mn^{2+}-activated inflammasome.

Supplementary Fig. 3. Mn^{2+} activates NLRP3 inflammasome in murine and human macrophages.

Supplementary Fig. 4. A formulation screening identifies MnJ adjuvant.

Supplementary Fig. 5. Properties of MnJ adjuvant.

Supplementary Fig. 6. Gating strategies of antigen presenting cells and germinal center cells.

Supplementary Fig. 7. Both cGAS-STING and NLRP3 contribute to adjuvant activity of MnJ.

Supplementary Fig. 8. MnJ enhances the protection efficacy of inactivated virus and subunit vaccine.

Supplementary Table 1. Formulations of manganese salts.

Supplementary Table 2. Information of peripheral blood mononuclear cell donors.

Supplementary Table 3. Target sequences of sgRNAs.

Supplementary Table 4. Primers for qRT-PCR.

References

Materials and Methods

Reagents

Mouse IL-1β ELISA Kit (MULTI SCIENCES, 70-EK201B2/2), Mouse IL-18 ELISA Kit (MULTI SCIENCES, 70-EK2182), Human IL-1β ELISA Kit (Invitrogen, 88-7261-22), Human IL-18 ELISA Kit (Sino Biological, SEK 10119), Mouse IFNγ ELISA Kit (MULTI SCIENCES, 70-EK280/3-96), Mouse IL-2 ELISA Kit (MULTI SCIENCES, 70-EK2022/2), LPS (Sigma, L4130), Mouse IL-4 Uncoated ELISA Kit (Invitrogen, 88-7044-22), Mouse IL-10 Uncoated ELISA Kit (Invitrogen, 88-7105-22), Z-VAD-FMK (Selleck, S7023), ABT-263 (Selleck, S1001), OVA peptides specific for I-Ab (ISQAVHAAHAEINEAGR) or H-2Kb (SIINFEKL) or control peptide (FAPGNYPAL) were synthesized by Scilight Biotechnology, N-acetyl-L-cysteine (Sigma, A7250), Reduced L-glutathione (Sigma, G4251), 2-Aminoethyl diphenylborinate (Sigma, D9754), NP-KLH (Santa Cruz, sc-396216), NP-BSA (Biosearch Technologies, N-5050H-10), Reactive Oxygen Species Assay Kit (Solarbio, CA1410), Anakinra (MCE, HY-108841), Etanercept (MCE, HY-108847).

Inflammasome activation

1×10^6 cells were plated in 12-well plate overnight and the medium was changed to opti-MEM with LPS (500 ng/ml) for 3 h. After that, the cells were treated with or without inhibitors for 1 h. Then cells were stimulated with indicated concentrations of Mn^{2+}, aluminum salts, ATP (Amresco, Cat# Amresco 0220), VACV (Western Reserve-Vvt7 strain), Salmonella, Ca^{2+} or Silica.

Mitochondrial DNA depletion

THP1 cells were cultured in RPMI (GIBCO) supplemented with 10% FBS (GIBCO), 2 mM L-glutamine, 100 μg/ml sodium pyruvate, 50 μg/ml uridine and 50 ng/ml ethidium bromide for 6 days as previously described [1]. Depletion of mtDNA was measured by Real-Time PCR of mtDNA versus genomic DNA.

Measurement of microelement

About 200 mg tissue samples were digested with 2 ml HNO_3 in a microwave digestion system. Then the amounts of Mn and Al were measured by Inductively Couples Plasma Mass Spectrometry (ICP-MS, Thermo X SERIES II) as previously described [2].

Flow cytometry analysis of the ratio of immune cells

Single cells were isolated from spleens and lymph nodes. The cells were divided into two parts. One part was stained by FITC labeled anti-mouse CD3 (Biolegend, Cat# 100305), PE labeled anti-mouse CD4 (Biolegend, Cat# 116005), APC/Cy7 labeled anti-mouse B220 (Biolegend, Cat# 103223), APC labeled anti-mouse CD8a (Biolegend, Cat# 100711), PE/Cy7 labeled anti-mouse CD11c (Biolegend, Cat# 117317), PerCP/Cy5.5 labeled anti-mouse Dec205 (Biolegend, Cat# 138207), BV421 labeled anti-mouse PDCA (Biolegend, Cat# 127023) to analyze $CD4^+$ T cell ($CD3^+$, $CD4^+$), $CD8^+$ T cell ($CD3^+$, $CD8^+$), $CD4^+$ DC ($CD8a^-$, $Dec205^-$, $CD11c^{hi}$, $CD4^+$), $CD8^+$ DC ($CD8a^+$, $Dec205^+$, $CD11c^{hi}$) or pDC ($B220^+$, $PDCA^+$, $CD11c^{int}$). The other part was stained by FITC labeled anti-mouse CD11b (Biolegend, Cat# 101205), PE labeled anti-mouse Gr1 (Biolegend, Cat# 108407), PE/Cy7 labeled anti-mouse CD115 (Biolegend, Cat# 135523), APC labeled anti-mouse F4/80 (Biolegend, Cat# 123115), APC/Cy7 labeled anti-mouse B220 (Biolegend, Cat# 103223), BV421 labeled anti-mouse NK1.1 (Biolegend, Cat# 108731) to

analyze macrophage ($CD11b^+$, $F4/80^+$), neutrophil ($CD11b^+$, $Gr1^+$), monocyte ($CD11b^+$, $CD115^+$), NK ($NK1.1^+$) or B cell ($B220^+$).

Ultra-structure observation

MnJ was mounted on 230-mesh copper grids, which were cleaned with ddH_2O for 3 times. Afterwards, the grids were dried overnight and observed under TEM (Ht-7700, Hitachi).

Tumor infiltrating T cell analysis

Tumor tissues were collected on day 14 after inoculation. For FACS analysis, the tissues were cut into pieces and incubated in a PBS solution containing Collagenase A (0.3 mg/ml) and DNase I (0.01 mg/ml) for 60 min at 37 °C under gentle rotation. Digestion was stopped by adding FBS on ice. The supernatants were centrifuged at 4000 rpm for 10 min at 4 °C. The samples were then resuspended in 1 ml PBS and filtered through a 100 mesh nylon sieve, followed by washing. Red blood cells were removed by ACK lysis buffer (155 mM NH_4Cl, 10 mM $KHCO_3$, 0.1 mM EDTA). Cells were incubated with APC labeled anti-CD4 (Biolegend, Cat# 100411) and PE/Cy7 labeled anti-CD8a (Biolegend, Cat# 100721). Tumor infiltrating T cells were analyzed by FACS. Fixed tumor tissues were embedded in 4% paraffin and cut into 4 μm sections. $CD4^+$ T cells were stained with anti-CD4 monoclonal antibody (Servicebio, GB13064-2) and FITC-labeled Goat Anti-Rabbit IgG (H+L) (Servicebio, GB22303). $CD8^+$ T cells were stained with anti-CD8 monoclonal antibody (Servicebio, GB11068) and Cy3 conjugated Goat Anti-rabbit IgG (H+L) (Servicebio, GB21303). Tissues were stained with DAPI (Servicebio, G1012) finally. Images were acquired by using a confocal microscope (Andor Dragonfly).

Tetramer staining

C57BL/6 mice were immunized with OVA (100 μg) and OVA (100 μg) + Mn^{2+} (20 μg) on day 0, 7 and 14. On day 21, splenocytes (1×10^6) from immunized mice were isolated and hemolyzed with ACK lysis buffer. Cells were incubated with PE labeled H2-Kb/OVA (SIINFEKL) tetramers (MBL, TS-5001-1C) for 30 min at 4 °C. Then add FITC labeled anti-CD8 (MBL, Cat#D271-4) to incubate for 30 min at 4 °C. After washing, cells were analyzed by FACS.

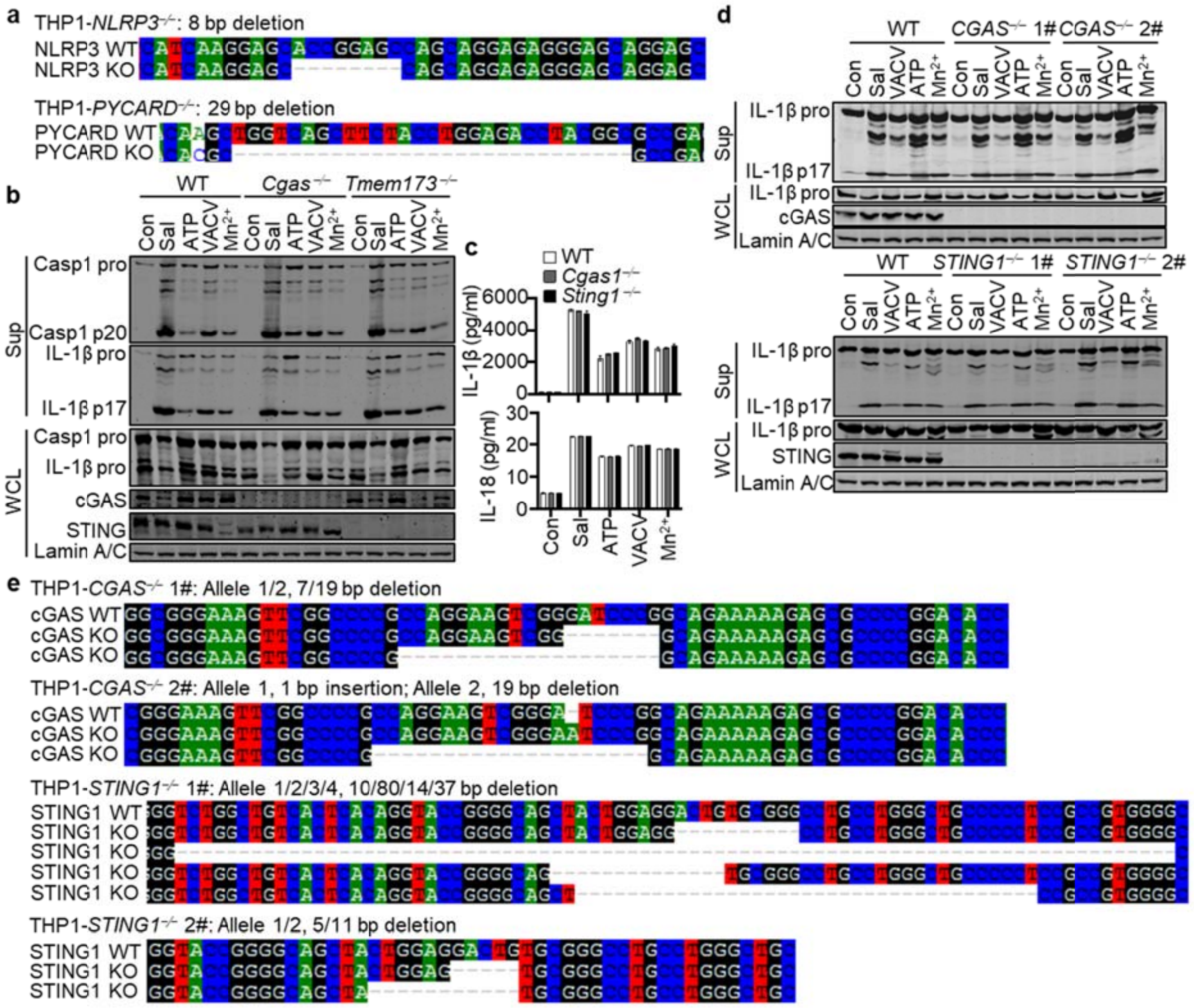

Supplementary Fig. 1. Mn^{2+} activates NLRP3 independent of cGAS or STING. a Genotyping of *NLRP3*$^{-/-}$ and *PYCARD*$^{-/-}$ THP1 cells. **b, c** LPS-primed WT, *Cgas*$^{-/-}$ and *Sting1*$^{-/-}$ peritoneal macrophages were stimulated with Salmonella (MOI = 10) or ATP (5 mM) for 1 h, VACV (MOI = 10) or Mn^{2+} (200 μM) for 5 h. Supernatants (Sup) and whole cell lysates (WCL) were analyzed by immunoblotting with the indicated antibodies **(b)**. IL-1β and IL-18 in supernatants were analyzed by ELISA **(c)**. Sal, *Salmonella*. **d** LPS-primed WT, *CGAS*$^{-/-}$ or *STING1*$^{-/-}$ THP1 cells were treated with Salmonella (MOI = 10), ATP (5 mM) and $MnCl_2$ (1 mM). The cleavage of IL-1β was analyzed by Western blot. **e** Genotyping of *CGAS*$^{-/-}$ and

STING$^{-/-}$ THP1 cells. One representative experiment of at least three independent experiments is shown, and each was done in triplicate.

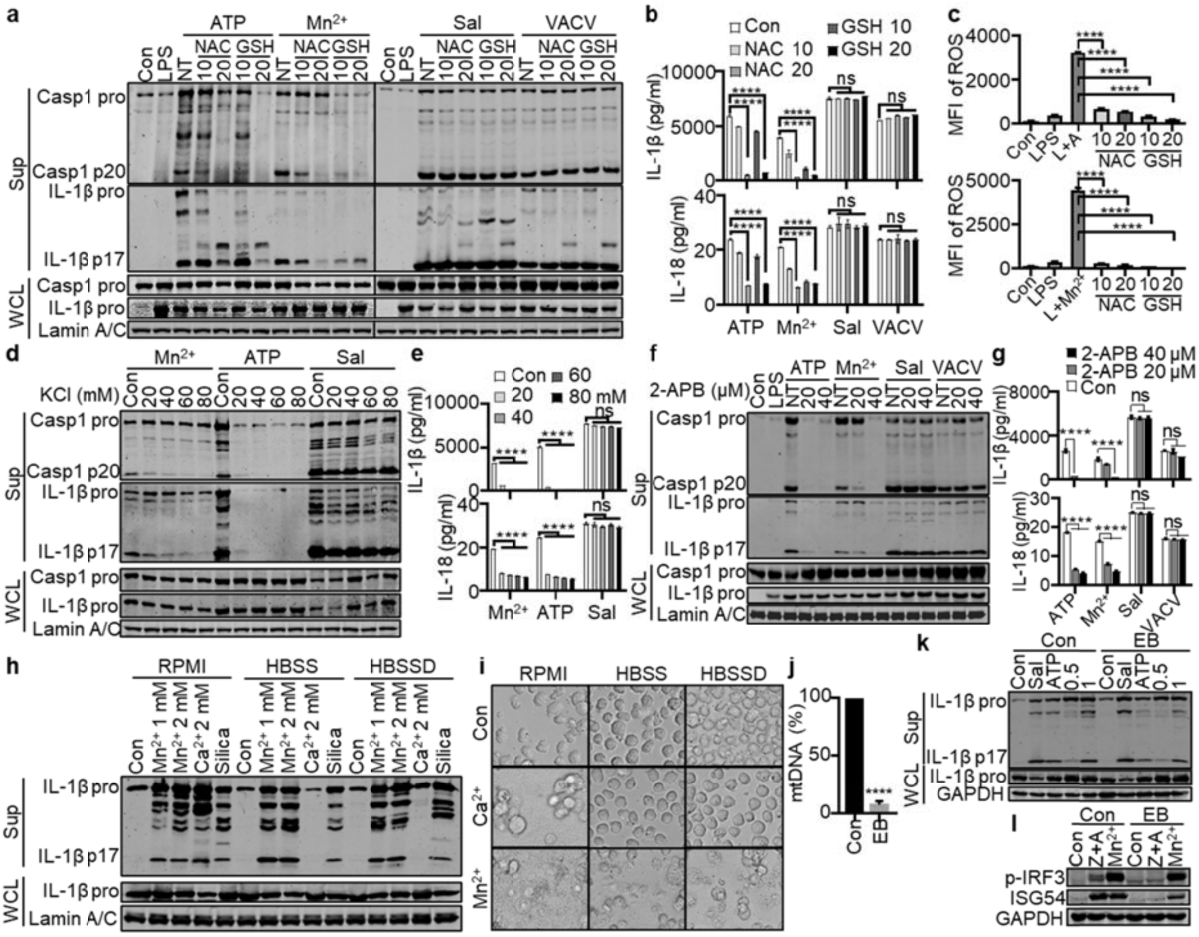

Supplementary Fig. 2. ROS, K^+ Efflux and Ca^{2+} release are required for Mn^{2+}-activated inflammasome. a, b Western blot **(a)** and ELISA analysis **(b)** of inflammasome activation of LPS-primed peritoneal macrophages treated with ATP (5 mM), $MnCl_2$ (0.5 mM), Salmonella (MOI = 10) or VACV (MOI = 10), which were pretreated with ROS inhibitor NAC (10 or 20 mM) or GSH (10 or 20 mM) for 1 h. **c** Intracellular ROS levels of cells in **(a)** were determined by FACS using DCFH-DA ROS probe. LPS (500 ng/ml), L + A, LPS (500 ng/ml) + ATP (5 mM). L + Mn^{2+}, LPS (500 ng/ml) + Mn^{2+} (0.5 mM). **d, e** Western blot **(d)** and ELISA analysis **(e)** of inflammasome activation of LPS-primed peritoneal macrophages treated with ATP (5 mM), $MnCl_2$ (0.5 mM) or Salmonella (MOI = 10), which were cultured in Opti-MEM with the

indicated extra concentrations of extracellular K^+. **f, g** Western blot **(f)** and ELISA analysis **(g)** of inflammasome activation of LPS-primed peritoneal macrophages treated with ATP (5 mM), Mn^{2+} (0.5 mM), Salmonella (MOI = 10) or VACV (MOI = 10), which were pretreated with Ca^{2+} release inhibitor 2-APB for 1 h. **h** Western blot analysis of inflammasome activation of LPS-primed THP1 cells treated with $MnCl_2$ (1 and 2 mM), $CaCl_2$ (2 mM) and Silica (0.1 mg/ml) for 5 h cultured in RPMI, HBSS (5.33 mM KCl, 0.44 mM KH_2PO_4, 4.17 mM $NaHCO_3$, 137.93 mM NaCl, 0.34 mM Na_2HPO_4, 5.56 mM D-Glucose) or HBSSD (5.33 mM KCl, 137.93 mM NaCl, 5.56 mM D-Glucose). **i** Images of pyroptosis of THP1 cells treated in **(h)**. **j** Quantitative PCR analysis of mtDNA versus genomic DNA from control and EB-co-cultured THP1 cells (n = 3). **k** Western blot analysis of inflammasome activation of LPS-primed control and EB-co-cultured THP1 cells treated with Salmonella (MOI = 10), ATP (5 mM) and $MnCl_2$ (0.5 and 1 mM). **l** Western blot analysis of Type I-IFN production in control and EB-co-cultured THP1 cells treated with Z-VAD-FMK (10 μM) + ABT263 (10 μM) or $MnCl_2$ (0.5 mM) for 18 h. Z+A, Z-VAD-FMK + ABT263. One representative experiment of at least three independent experiments is shown, and each was done in triplicate. Error bars represent SEM; (**b, c, e, g, j)** data were analyzed by an unpaired t test. ns, not significant; * $P < 0.05$; ** $P < 0.01$; *** $P<0.001$; **** $P < 0.0001$.

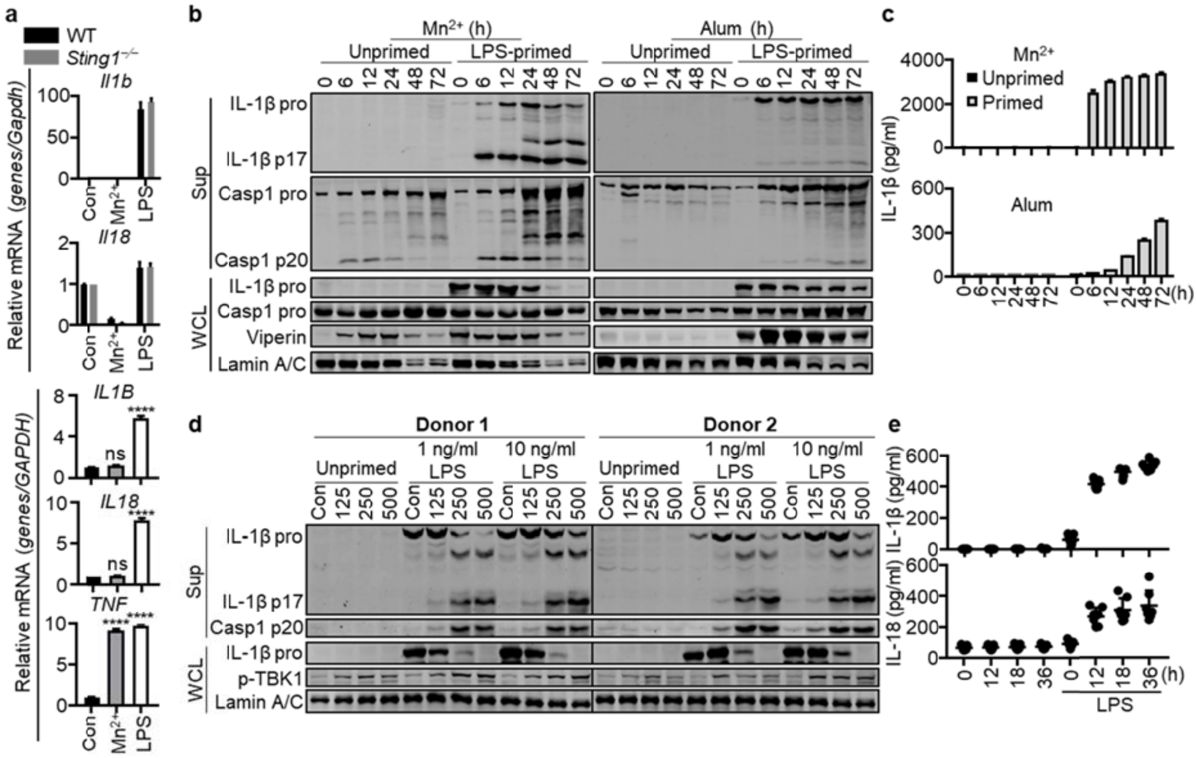

Supplementary Fig. 3. Mn^{2+} activates NLRP3 inflammasome in murine and human macrophages. **a** Quantitative RT-PCR analysis of the indicated gene expression in the WT and *Sting1*$^{-/-}$ BMDCs (upper) or human Mo-DCs (lower) treated with $MnCl_2$ (200 μM) or LPS (100 ng/ml) for 20 h. **b, c** Unprimed or LPS-primed mouse peritoneal macrophages were treated with $MnCl_2$ (200 μM) or Imject Alum (100 μg/ml) for the indicated times. Supernatants (Sup) and whole cell lysates (WCL) were analyzed by immunoblotting with the indicated antibodies **(b)**. IL-1β in supernatants was analyzed by ELISA **(c)**. **d** Unprimed or LPS (1 ng/ml or 10 ng/ml)-primed human PBMCs were treated with $MnCl_2$ (125, 250 or 500 μM) for 12 h. Supernatants and whole cell lysates were analyzed by immunoblotting with the indicated antibodies **e** Unprimed or LPS (1 ng/ml)-primed human PBMCs were treated with $MnCl_2$ (200 μM) for the indicated times. IL-1β and IL-18 in supernatants were analyzed by ELISA (n = 7). One representative experiment of at least three independent experiments is shown, and each was done

in triplicate. Error bars represent SEM; **(a)** data were analyzed by an unpaired t test. ns, not significant; * $P < 0.05$; ** $P < 0.01$; *** $P < 0.001$; **** $P < 0.0001$.

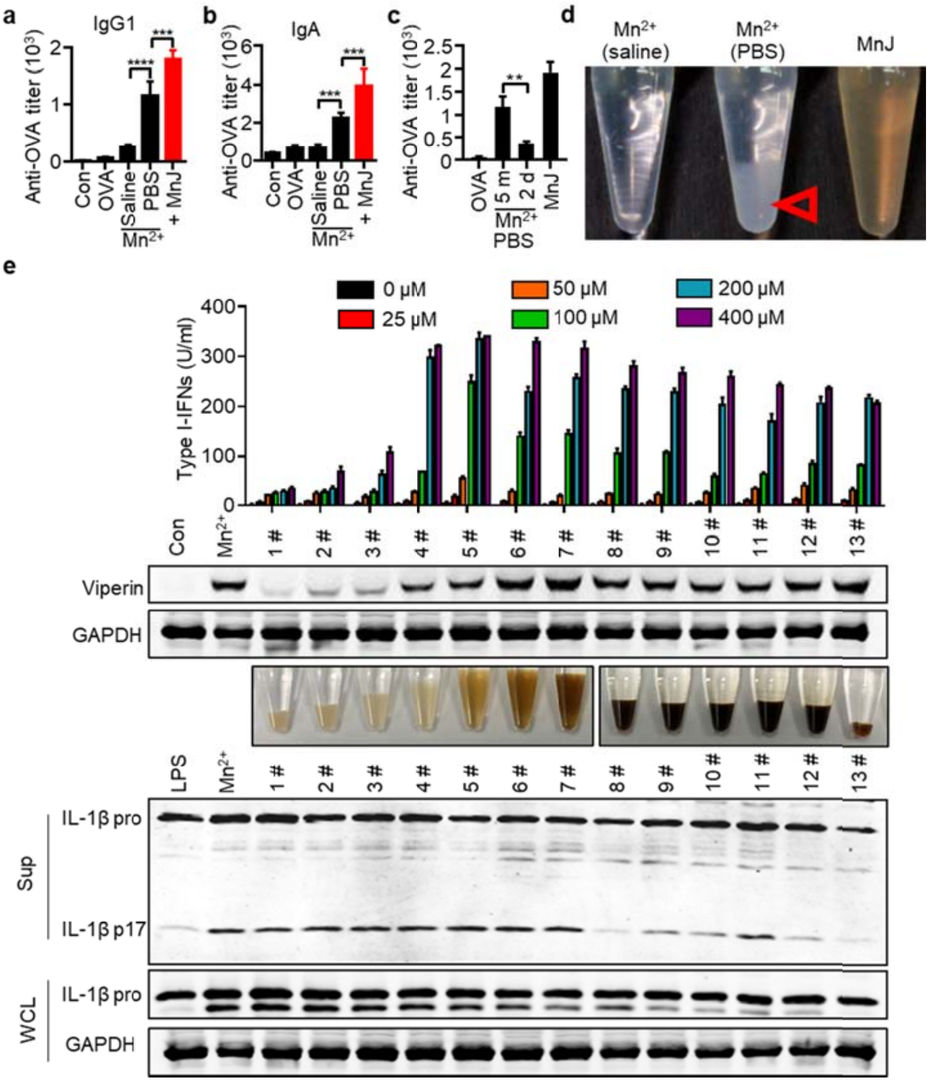

Supplementary Fig. 4. A formulation screening identifies MnJ adjuvant. a The WT mice were immunized intramuscularly with PBS, OVA (10 μg), OVA (10 μg) + Mn^{2+} (10 μg in normal saline), OVA (10 μg) + Mn^{2+} (10 μg in PBS) or OVA (10 μg) + MnJ (10 μg in normal saline) on day 0, 7 and 14. Sera were collected on day 21 to measure OVA-specific IgG1 by ELISA (n = 3). **b** The WT mice were immunized intranasally with PBS, OVA (10 μg), OVA (10 μg) + Mn^{2+} (5 μg in normal saline), OVA (10 μg) + Mn^{2+} (5 μg in PBS) or OVA (10 μg) + MnJ (5 μg in normal saline) on day 0, 7 and 14. Sera were collected on day 21 to measure OVA-specific IgA by ELISA (n = 3). **c** The WT mice were immunized intramuscularly with OVA (10

μg), OVA (10 μg) + Mn^{2+} (10 μg in PBS for 5 min), OVA (10 μg) + Mn^{2+} (10 μg in PBS for 2 d) or OVA (10 μg) + MnJ (10 μg in normal saline) on day 0, 7 and 14. Sera were collected on day 21 to measure OVA-specific IgG1 by ELISA (n = 3). **d** $MnCl_2$ (20 mM each) in normal saline, PBS or saline containing 25 mM Na_3PO_4 (MnJ), mixtures were settled overnight before pictures were taken. Aggregated and precipitated Mn salts in PBS was indicated by an open arrow. **e** Manganese salts were generated by mixing $MnCl_2$/NaOH/Na_3PO_4 with final concentrations in Supplementary Table 1 and continuously stirred for 8 h at 500 rpm. Particles in the suspension were collected by centrifugation at 1000 × g for 30 min. The pellets were suspended in normal saline. THP1 cells were treated with indicated concentrations of manganese salts for 24 h. The production of Type I-IFN was measured by bioassay. The expression of Viperin was analyzed by western blot. LPS-primed THP1 cells were treated with $MnCl_2$ or manganese salts (1 mM) for 5 h. The cleavage of IL-1β was measured by western blot. One representative experiment of at least three independent experiments is shown, and each was done in triplicate. Error bars represent SEM. ns, not significant; * $P < 0.05$; ** $P < 0.01$; *** $P<0.001$; **** $P < 0.0001$.

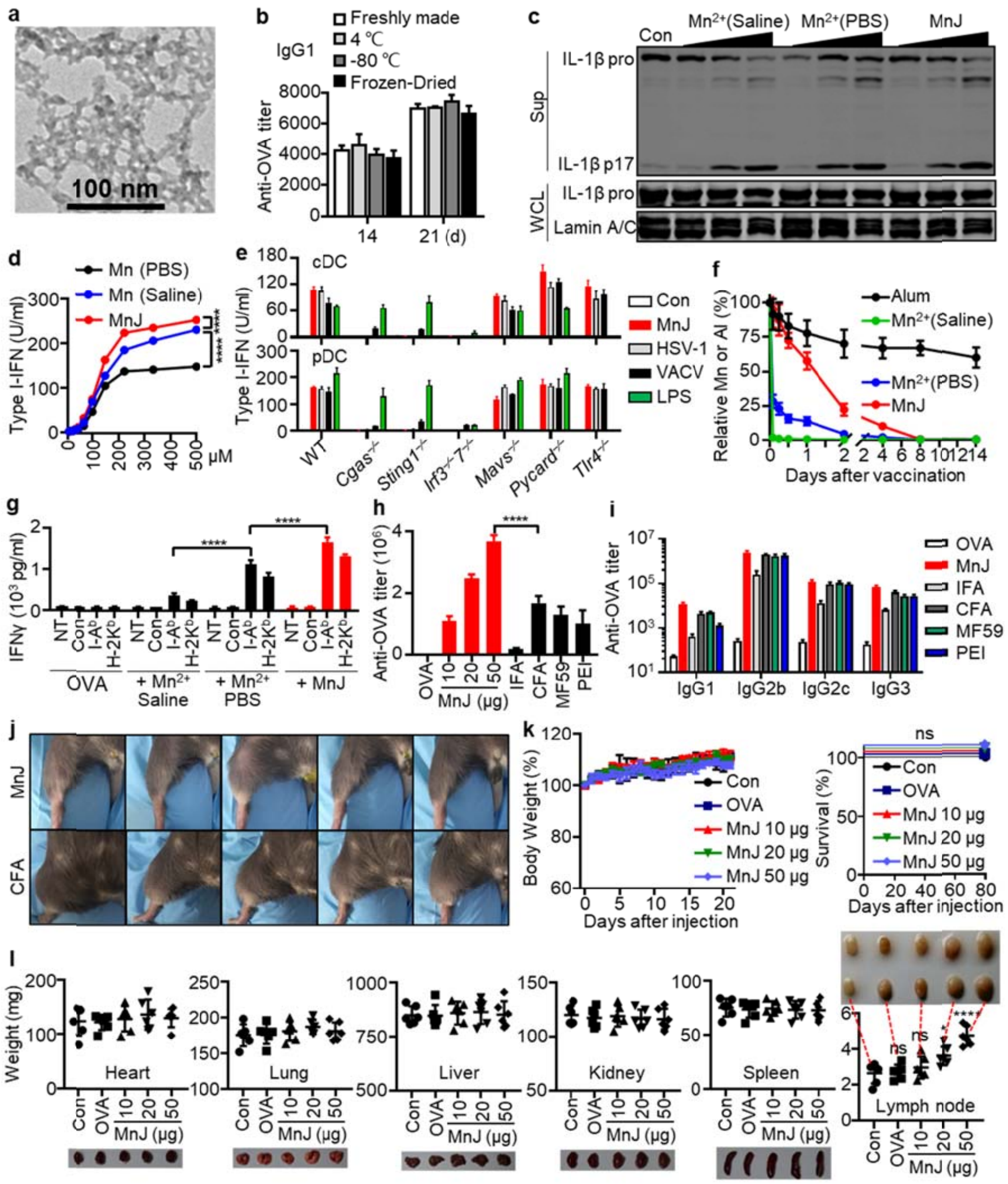

Supplementary Fig. 5. Properties of MnJ adjuvant. a Representative image of MnJ nanoparticles. Images were obtained by TEM. Scale bar = 100 nm. **b** The WT mice were immunized intramuscularly with OVA (10 μg) + MnJ (10 μg freshly made), OVA (10 μg) +

MnJ (10 μg kept at 4 ºC), OVA (10 μg) + MnJ (10 μg kept at -80 ºC) or OVA (10 μg) + MnJ (10 μg frozen-dried) on day 0, 7 and 14. Sera were collected on day 21 to measure OVA-specific IgG1 by ELISA (n = 8). **c** LPS-primed THP1 cells in HBSSD (5.33 mM KCl, 137.93 mM NaCl, 5.56 mM D-Glucose) were treated with $MnCl_2$ + normal saline, $MnCl_2$ + PBS or MnJ for 5 h. The cleavage of IL-1β was measured by Western blot. All Mn^{2+} stock solutions contain 20 mM Mn^{2+} and cells were treated with 250, 500 and 1000 μM Mn^{2+}. **d** THP1 cells were treated with $MnCl_2$ + normal saline, $MnCl_2$ + PBS or MnJ for 24 h. All Mn^{2+} stock solutions contain 20 mM Mn^{2+} and cells were treated with 6, 9, 13, 20, 29, 44, 66, 99, 148, 222, 333 and 500 μM Mn^{2+}. The Production of Type I-IFN was measured by bioassay. **e** Type I-IFN activity in the culture medium of WT and indicated gene-deficient cDCs and pDCs treated with MnJ, HSV-1, VACV and LPS for 24 h. **f** The WT mice were injected intramuscularly with 100 μg Imject Alum, 20 μg $MnCl_2$ (in normal saline), 20 μg $MnCl_2$ (in PBS) or 20 μg MnJ. Muscles at the injection site (100 mg) were collected at the indicated times to measure the amounts of Mn and Al by ICP-MS (n = 3). **g** The WT mice were injected intramuscularly with OVA (10 μg), OVA (10 μg) + Mn^{2+} (10 μg in normal saline), OVA (10 μg) + Mn^{2+} (10 μg in PBS) or OVA (10 μg) + MnJ (10 μg in normal saline) on day 0, 7 and 14. Splenocytes were collected on day 21, and stimulated with OVA peptides. IFNγ secreted by T cells was measured by ELISA. **h, i** OVA-specific total IgG, IgG1, IgG2b, IgG2c, IgG3 were measured by ELISA on day 21 after immunization with OVA (10 μg), OVA (10 μg) + indicated amounts of MnJ, IFA (50 μl), CFA (50 μl), MF59 (50 μl) or PEI (100 μg) intramuscularly on day 0, 7 and 14 (n = 3). **j** Swelling and granulomas formed at the site of injection from mice (n = 5) immunized intramuscularly with CFA (50 μl CFA + 50 μl normal saline) after one injection (bottom) or mice were immunized intramuscularly with 50 μg MnJ (in 100 μl normal saline, once a week) after three injections (Top). Photos were taken on

day 7 (CFA) or day 21 (MnJ) respectively. **k** The WT mice were immunized intramuscularly with OVA (10 μg) + MnJ (10, 20 and 50 μg) on day 0, 7 and 14. Their body weight (left) and survival (right) were monitored for 3 weeks. **l** On day 40, hearts, lungs, livers, kidneys, spleens and inguinal lymph nodes were collected and organ weights were recorded (n = 6). One representative experiment of at least three independent experiments is shown, and each was done in triplicate. Error bars represent SEM; **(g, h, l)** data were analyzed by an unpaired t test; **(d)** data were analyzed by two-way ANOVA; **(k)** survival plot data were analyzed with log-rank (Mantel–Cox) tests. ns, not significant.

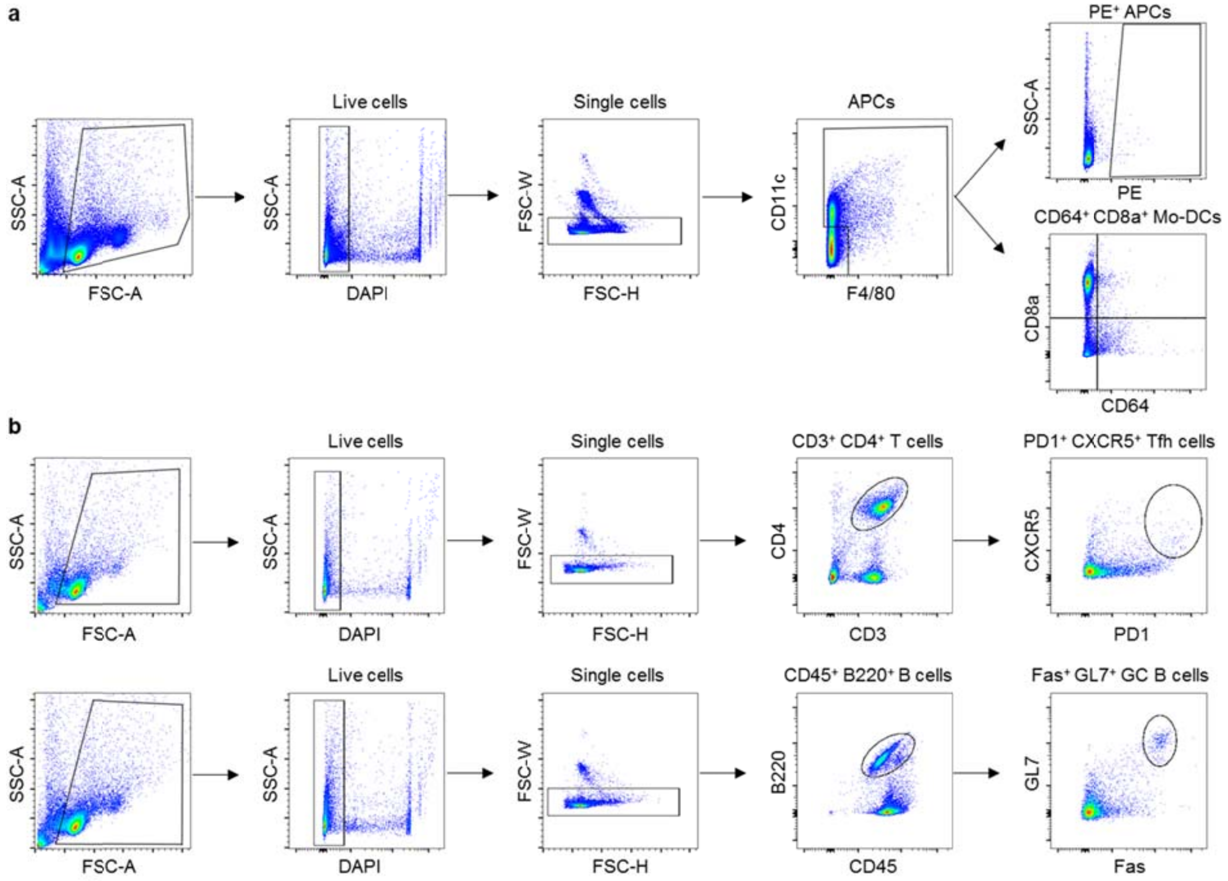

Supplementary Fig. 6. Gating strategies of antigen presenting cells and germinal center cells. a Exemplary gating strategy for detecting PE^+ APCs and Mo-DCs. Plots are pre-gated on $F4/80^+$ or $CD11c^+$ APCs and further separated based on the expression of PE, CD64 and CD8a. **b** Exemplary gating strategy for detecting Tfh and GC B cells. Plots are pre-gated on $CD3^+$ $CD4^+$ T cells or $CD45^+$ $B220^+$ B cells and further separated based on the expression of PD1, CXCR5, Fas and GL7.

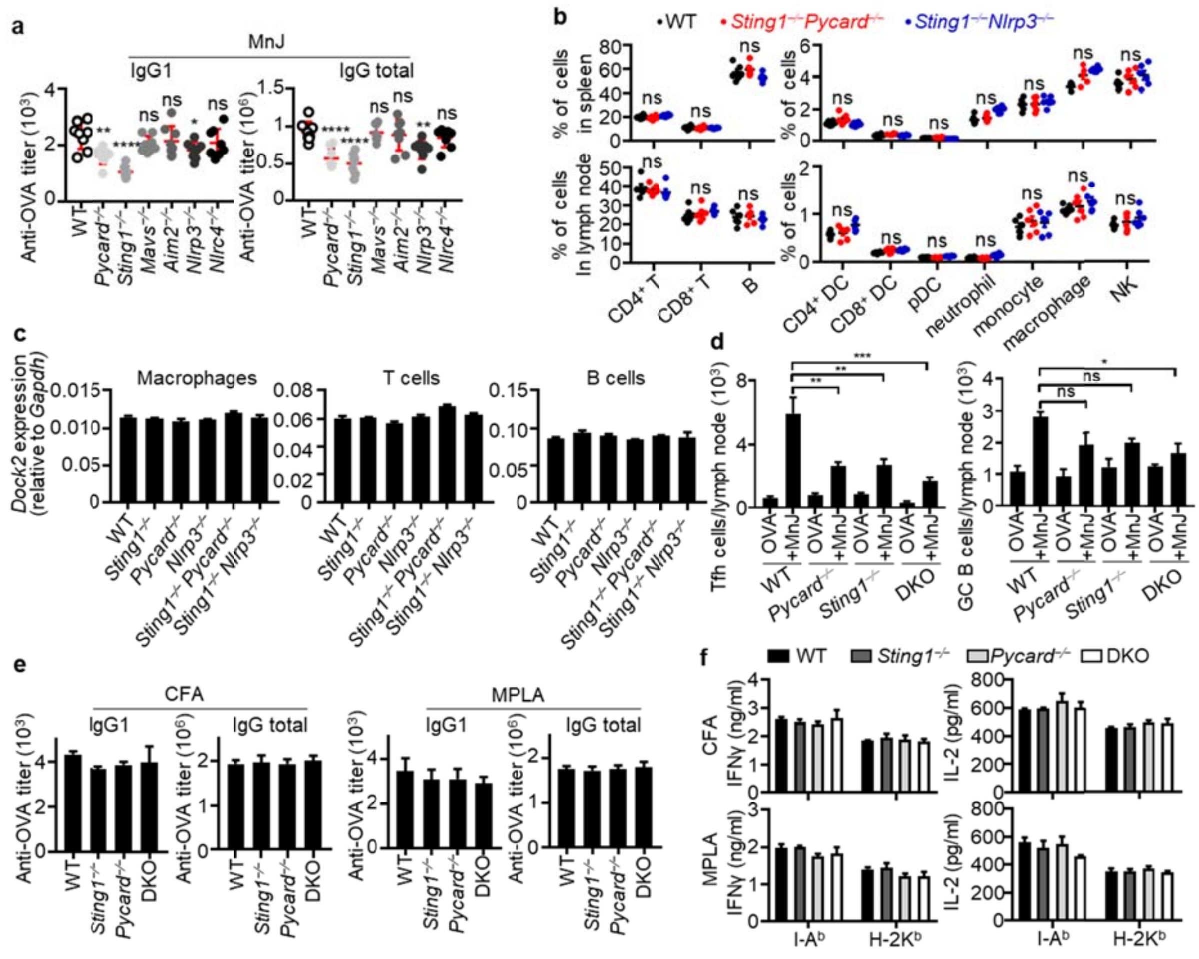

Supplementary Fig. 7. Both cGAS-STING and NLRP3 contribute to adjuvant activity of MnJ. a The WT, *Pycard*$^{-/-}$, *Sting1*$^{-/-}$, *Mavs*$^{-/-}$, *Aim2*$^{-/-}$, *Nlrp3*$^{-/-}$ and *Nlrc4*$^{-/-}$ mice were immunized intramuscularly with OVA (10 μg) + MnJ (10 μg) on day 0, 7 and 14. Sera were collected on day 21 to measure OVA-specific IgG1 and IgG total by ELISA (n = 8). **b** The percentage of immune cells in spleens and lymph nodes of the WT, *Sting1*$^{-/-}$ *Pycard*$^{-/-}$ and *Sting1*$^{-/-}$ *Nlrp3*$^{-/-}$ mice. **c** Quantitative RT-PCR analysis of *Dock2* expression in the peritoneal macrophages, T cells and B cells of WT, *Sting1*$^{-/-}$, *Pycard*$^{-/-}$, *Nlrp3*$^{-/-}$, *Sting1*$^{-/-}$ *Pycard*$^{-/-}$ and *Sting1*$^{-/-}$ *Nlrp3*$^{-/-}$ mice. **d** Numbers of Tfh or GC B cells in dLN from WT, *Pycard*$^{-/-}$, *Sting1*$^{-/-}$, DKO mice were analyzed by FACS. Live cells were identified by DAPI staining. Among live

singlet cells, $CD4^+$ T cells were identified as the cell subset double positive for CD3 and CD4. Among $CD4^+$ T cells, Tfh cells were identified as $PD1^+$ $CXCR5^+$ cells. B cells were identified as the cell subset double positive for CD45 and B220. Among B cells, GC B cells were identified as Fas^+ $GL7^+$ cells. **e, f** The WT, *Sting1*$^{-/-}$, *Pycard*$^{-/-}$ and DKO mice were immunized intramuscularly with OVA (10 μg) + CFA (50 μl) or MPLA (2 μl) on day 0, 7 and 14. Sera were collected on day 21 to measure OVA-specific IgG1 and IgG total by ELISA (n = 3) **(e)**. Splenocytes of the immunized mice were stimulated by I-A^b and H-2K^b for 72 h. IL-4 and IL-10 were measured by ELISA **(f)**. MPLA was SIGMA ADJUVANT SYSTEM (R) (S6322-1VL). One representative experiment of at least three independent experiments is shown, and each was done in triplicate. Error bars represent SEM; **(a, b, d)** data were analyzed by an unpaired t test. ns, not significant; * $P < 0.05$; ** $P < 0.01$; *** $P<0.001$; **** $P < 0.0001$.

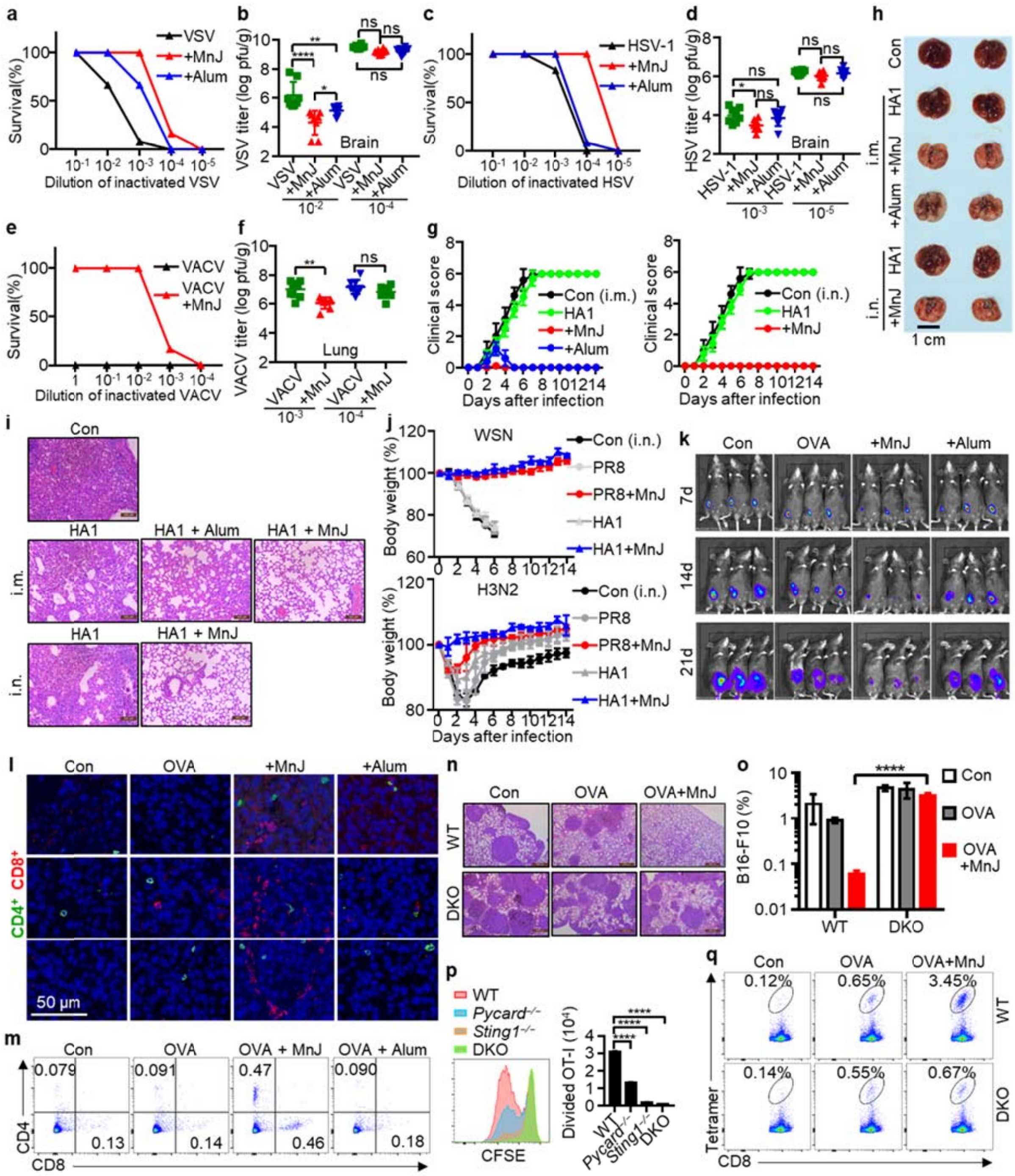

Supplementary Fig. 8. MnJ enhances the protection efficacy of inactivated virus and subunit vaccine. a, b The WT mice were immunized intramuscularly with PBS, inactivated 10^{-1} VSV (1×10^{7} pfu), 10^{-2} VSV (1×10^{6} pfu), 10^{-3} VSV (1×10^{5} pfu), 10^{-4} VSV (1×10^{4} pfu) or

10^{-5} VSV (1×10^3 pfu) with or without MnJ (10 μg) and Imject Alum (1320 μg) on day 0. On day 10, these mice were infected intravenously with a lethal dose of VSV. The survival was monitored for 2 weeks (n = 12) **(a)**. Viral loads in brain were measured 5 days after infection (n = 8) **(b)**. **c, d** The WT mice were immunized intramuscularly with PBS, inactivated 10^{-1} HSV-1 (1×10^6 pfu), 10^{-2} HSV-1 (1×10^5 pfu), 10^{-3} HSV-1 (1×10^4 pfu), 10^{-4} HSV-1 (1×10^3 pfu) or 10^{-5} HSV-1 (1×10^2 pfu) with or without MnJ (10 μg) and Imject Alum (1320 μg) on day 0. On day 10, these mice were infected intraperitoneally with a lethal dose of HSV-1. The survival was monitored for 2 weeks (n = 12) **(c)**. Viral loads in brain were measured 5 days after infection (n = 8) **(d)**. **e, f** The WT mice were immunized intranasally with PBS, inactivated VACV (2×10^6 pfu), 10^{-1} VACV (2×10^5 pfu), 10^{-2} VACV (2×10^4 pfu), 10^{-3} VACV (2×10^3 pfu) or 10^{-4} VACV (2×10^2 pfu) with or without MnJ (5 μg) on day 0 and 7. On day 14, these mice were infected intranasally with a lethal dose of VACV. The survival was monitored for 2 weeks (n = 12) **(e)**. Viral loads in lung were measured 5 days after infection (n = 8) **(f)**. **g** Clinical scores of mice in Figure 7G were daily evaluated for 2 weeks. **h** Images of lungs from mice treated in Figure 7G were recorded on day 5. **i** HE-stained lung sections from mice in Figure 7G were analyzed on day 5 by Leica CTR5000. **j** The WT mice were immunized intranasally with PBS, inactivated PR8 (5×10^6 pfu), inactivated PR8 (5×10^6 pfu) + MnJ (10 μg) for 2 times or HA1 (5 μg) or HA1 (5 μg) + MnJ (10 μg) for 3 times. Then, these mice were infected intranasally with a lethal dose of H1N1 WSN subtype A/WSN/1933 and H3N2 subtype A/Jiangxi/262/2005. Body weight was recorded for 2 weeks (n = 5). **k-m** The WT mice were immunized intramuscularly with PBS, OVA (10 μg), OVA (10 μg) + MnJ (20 μg) or OVA (10 μg) + Imject Alum (1320 μg) on day 0, 7 and 14. On day 21, these mice were inoculated with B16-OVA-Fluc cells (3×10^5) subcutaneously. Representative IVIS images were acquired on day 7, 14 and 21

after inoculation **(k)**. Some tumors were collected on day 14 and immune-staining of $CD4^+$ and $CD8^+$ T cells in tumor sections were recorded **(l)**. Tumor infiltrating T cells were analyzed by FACS on day 14 **(m)**. **n** HE-stained lung sections from mice in Figure 7L were analyzed. **o** The WT and DKO mice were immunized intramuscularly with PBS, OVA (10 μg), or OVA (10 μg) + MnJ (20 μg) on day 0, 7 and 14. On day 21, these mice were inoculated with B16-F10-OVA-GFP (3×10^5) intravenously. Percentage of GFP^+ B16-F10 in lung was analyzed by FACS on day 20 after inoculation. **p** $CD45.1^+$ OT-I $CD8^+$ T cells were labeled with CFSE and transferred to $CD45.2^+$ WT, *Sting1*$^{-/-}$, *Pycard*$^{-/-}$ and DKO mice. These mice were then immunized with OVA (1 μg) + MnJ (10 μg). After 3 days, T cell proliferation was analyzed by FACS (n = 3). **q** The WT and *Sting1*$^{-/-}$*Pycard*$^{-/-}$ DKO mice were immunized intramuscularly with PBS, OVA (10 μg), OVA (10 μg) + MnJ (20 μg) on day 0, 7 and 14. On day 21, the percentage of $tetramer^+$ $CD8^+$ T cells in spleens of these mice was analyzed by FACS. One representative experiment of at least three independent experiments is shown, and each was done in triplicate. Error bars represent SEM; **(b, d, f, o, p)** data were analyzed by an unpaired t test. ns, not significant; * $P < 0.05$; ** $P < 0.01$; *** $P<0.001$; **** $P < 0.0001$.

Supplementary Table 1. Formulations of manganese salts.

Manganese salts	Mn^{2+} (mM)	OH^{-} (mM)	PO_4^{3-} (mM)
1 #	10	0	6.67
2 #	10	0.5	6.5
3 #	10	1	6.33
4 #	10	2	6
5 #	10	4	5.33
6 #	10	6	4.67
7 #	10	8	4
8 #	10	10	3.33
9 #	10	12	2.67
10 #	10	14	2
11 #	10	16	1.33
12 #	10	18	0.66
13 #	10	20	0

Supplementary Table 2. Information of peripheral blood mononuclear cell donors

Number	Age	Sex	Physical condition
1	26	Male	Healthy
2	22	Male	Healthy
3	22	Female	Healthy
4	25	Female	Healthy
5	23	Female	Healthy
6	21	Female	Healthy
7	24	Female	Healthy

Supplementary Table 3. Target sequences of sgRNAs

Target of sgRNA	Sequence
cGAS	5’-CCGCCAGGAAGTCGGGATCC-3’
STING	5’-CAGCTACTGGAGGACTGTGC-3’
NLRP3	5’-TGCGTCTCATCAAGGAGCAC-3’
PYCARD	5’-CAAGCTGGTCAGCTTCTACC-3’

Supplementary Table 4. Primers for qRT-PCR

Primers	Forward (5'- 3')	Reverse (5'- 3')
Ifnb1	GCCTTTGCCATCCAAGAGATGC	ACACTGTCTGCTGGTGGAGTTC
Ifna1	GGATGTGACCTTCCTCAGACTC	ACCTTCTCCTGCGGGAATCCAA
Ifna2	ATCCAGAAGGCTCAAGCCATCC	GGAGGGTTGTATTCCAAGCAGC
Ifna4	GCAATGACCTCCATCAGCAGCT	GTGGAAGTATGTCCTCACAGCC
Ifit1	TACAGGCTGGAGTGTGCTGAGA	CTCCACTTTCAGAGCCTTCGCA
Ifit2	CGAACTACCGTCTGGATGACTG	CTTCAACCAGCGCCATTGCTTG
Ifit3	GCTCAGGCTTACGTTGACAAGG	CTTTAGGCGTGTCCATCCTTCC
Ccl4	ACCCTCCCACTTCCTGCTGTTT	CTGTCTGCCTCTTTTGGTCAGG
Ccl5	CCTGCTGCTTTGCCTACCTCTC	ACACACTTGGCGGTTCCTTCGA
Il6	TACCACTTCACAAGTCGGAGGC	CTGCAAGTGCATCATCGTTGTTC
Il10	CGGGAAGACAATAACTGCACCC	CGGTTAGCAGTATGTTGTCCAGC
Il12a	ACGAGAGTTGCCTGGCTACTAG	CCTCATAGATGCTACCAAGGCAC
Il1b	TGGACCTTCCAGGATGAGGACA	GTTCATCTCGGAGCCTGTAGTG
Il18	GACAGCCTGTGTTCGAGGATATG	TGTTCTTACAGGAGAGGGTAGAC
Isg15	CATCCTGGTGAGGAACGAAAGG	CTCAGCCAGAACTGGTCTTCGT
Rsad2	GGAAGGTTTTCCAGTGCCTCCT	ACAGGACACCTCTTTGTGACGC
Tnfa	GGTGCCTATGTCTCAGCCTCTT	GCCATAGAACTGATGAGAGGGAG
Dock2	TTGCTCAGCCAGCTACTGTATG	TTGGTGATGACAGGAAGCAGAAT
Gapdh	CATCACTGCCACCCAGAAGACTG	ATGCCAGTGAGCTTCCCGTTCAG
IL1B	CCACAGACCTTCCAGGAGAATG	GTGCAGTTCAGTGATCGTACAGG
IL18	GATAGCCAGCCTAGAGGTATGG	CCTTGATGTTATCAGGAGGATTCA
TNFA	CTCTTCTGCCTGCTGCACTTTG	ATGGGCTACAGGCTTGTCACTC
GAPDH	GTCTCCTCTGACTTCAACAGCG	ACCACCCTGTTGCTGTAGCCAA
gDNA (GAPDH)	CTGTTCGACAGTCAGCCGCATC	GCGCCCAATACGAC CAAATCCG
mtDNA (COXII)	CCCCACATTAGGCTTAAAAACAGAT	TATACCCCCGGTCGTGTAGC

References:

1 Hashiguchi, K. & Zhang-Akiyama, Q. M. Establishment of human cell lines lacking mitochondrial DNA. *Methods Mol Biol* **554**, 383-391, doi:10.1007/978-1-59745-521-3_23 (2009).

2 Wang, C. *et al.* Manganese Increases the Sensitivity of the cGAS-STING Pathway for Double-Stranded DNA and Is Required for the Host Defense against DNA Viruses. *Immunity* **48**, 675-687 e677, doi:10.1016/j.immuni.2018.03.017 (2018).
